# Engineering high-fidelity bone organoids: Operational classification, multilineage crosstalk, biofabrication evidence, and translational validation

**DOI:** 10.1016/j.mtbio.2026.103676

**Published:** 2026-09-17

**Authors:** Yining Huang, Tianlong Zhang, Shuying Chen, Hengbing Zhou, Haocheng Xu, Fan Zhang, Linli Li, Feizhou Lyu

**Affiliations:** a Department of Orthopedics, Huashan Hospital, Fudan University, Shanghai, China; b Department of Orthopedics, Shanghai Fifth People's Hospital, Fudan University, China; c Department of Laboratory Medicine, Huashan Hospital, Fudan University, China

**Keywords:** Bone organoid, 3D bioprinting, Bone remodeling, Neurovascular coupling, Translational validation

## Abstract

Bone organoids are emerging as three-dimensional models of skeletal development, disease and regeneration. The term bone organoid is applied inconsistently to osteogenic spheroids, scaffold-dominated constructs and self-organizing skeletal tissues. This review establishes an operational five-class framework that distinguishes osteogenic spheroids, bone-like microtissues, engineered skeletal constructs, bone organoids and high-fidelity bone organoids. Core organoid criteria are separated from advanced, application-dependent features, and a structured evidence map applies the terminology to representative original studies. We summarize the coordinated osteogenic, chondrogenic, vascular, neural and immune programs that govern bone formation and examine how cell source, induction sequence, matrix composition, mass transport and mechanical stimulation affect maturation. Inkjet, extrusion, laser-assisted and photocuring-based bioprinting are compared using common technical and biological criteria, including resolution, viscosity, cell density, injury mechanisms, mineral compatibility, perfusable channels, scalability and direct bone-organoid evidence. Current data show that printing reliably controls initial geometry, but rarely demonstrates improved self-organization, multilineage interaction or long-term function relative to composition-matched controls. Translational requirements are therefore evaluated separately for developmental and genetic disease, metabolic and inflammatory disease, tumor–bone interactions, drug screening and regenerative grafts. We also define material-aware controls for active mineralization and scale-resolved mechanical testing. Available models reproduce important subsets of bone biology; however, among the representative studies examined, no single platform yet demonstrates hierarchical matrix maturation, coupled formation-resorption and controlled mechanosensitivity in combination. These evidence thresholds provide a practical basis for terminology, benchmarking and future translation.

## Highlights

• Defines a five-class framework for high-fidelity bone organoids.
• Integrates osteogenic, osteoclastic, vascular, neural, immune and chondrogenic axes.
• Compares top-down, bottom-up, hybrid and organoid-on-chip engineering strategies.
• Evaluates bioprinting using organoid-specific evidence and validation criteria.
• Links bone organoids to disease modeling, drug screening and regenerative grafts.

## Graphical abstract

**Figure:**
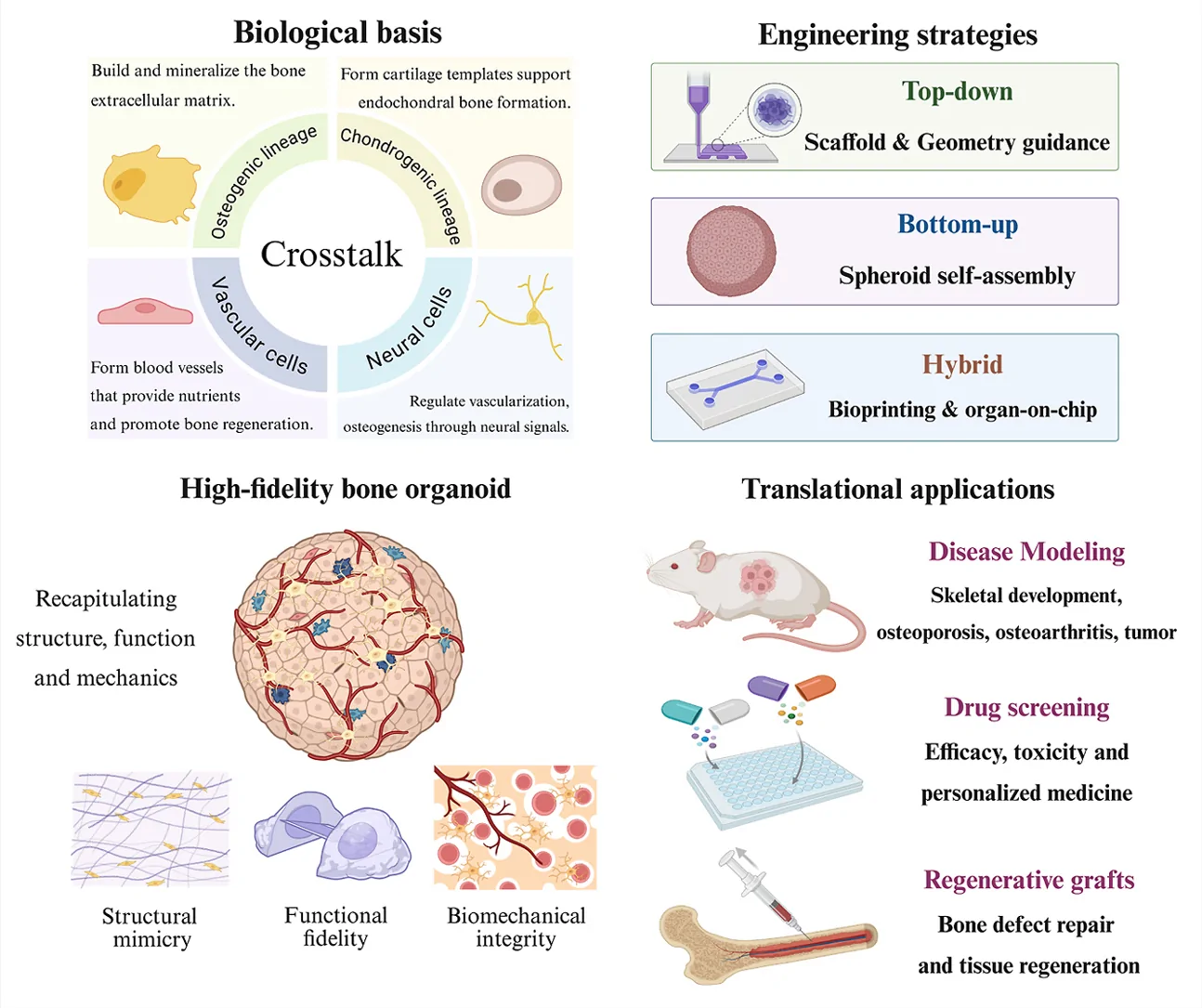

## 1 Introduction

Organoid technology bridges reductionist cell culture and clinically relevant tissue models. Patient-derived organoids can retain disease-specific phenotypes in three-dimensional culture and support personalized drug-response testing [[1], [2], [3]]. More broadly, organoids are cell-derived three-dimensional structures that acquire tissue-like organization through self-organization, externally imposed guidance or a combination of both [4,5]. Their value lies not simply in three-dimensionality, but in the sustained interaction of cells with extracellular matrix (ECM), soluble signals and physical constraints. Compared with two-dimensional monolayers, organoids better reproduce cell polarity, matrix contact, diffusion gradients and cell-cell communication; compared with animal models, they are more experimentally accessible and, when generated from human cells, reduce uncertainty arising from species-specific biology. Three-dimensional bioprinting extends this concept by enabling controlled placement of cell populations, matrices and channels within a construct [[6], [7], [8]]. Such spatial control is particularly relevant to bone, whose function depends on hierarchical architecture rather than osteogenic differentiation alone. However, current bone-organoid models vary markedly in cellular composition, degree of mineralization, structural organization and reproducibility, underscoring the need for precise terminology and transparent reporting.

The conceptual roots of organoid research extend to early twentieth-century experiments showing that dissociated cells can reaggregate and recover features of tissue organization [9]. The isolation of mouse embryonic stem cells in 1981 subsequently provided a renewable cell source with stable self-renewal and multilineage potential [10]. A major advance came in 2009, when Clevers and colleagues generated crypt-villus structures from single Lgr5-positive intestinal stem cells, demonstrating that a defined niche can support self-organization and tissue-specific function in the absence of an intact organ [11]. Similar approaches were later adapted to the liver, stomach, brain, lung, kidney and other tissues. Bone, however, presents a distinct engineering challenge: it is mineralized, mechanically active, continuously remodeled, vascularized and innervated (Fig. 1). Methods developed for soft epithelial organoids therefore cannot be transferred directly without accounting for stiffness, load transmission, oxygen delivery and the temporal transition from immature matrix to mineralized tissue.

**Fig. 1:**
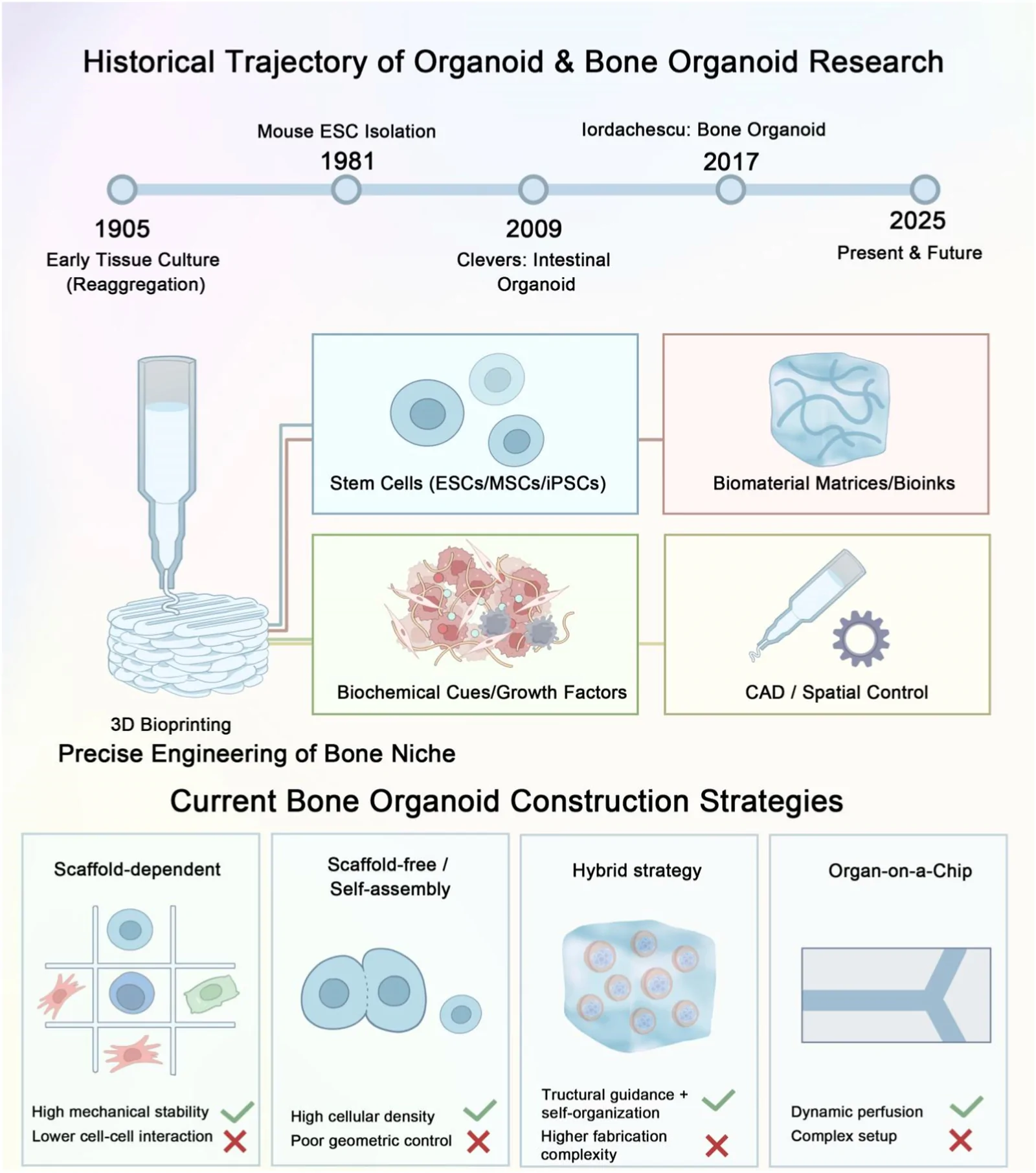
Historical trajectory and current strategies for bone-organoid engineering.

Within this broader context, the term “bone organoid” has increasingly been applied to sophisticated three-dimensional skeletal models. Iordachescu and colleagues helped shift the field from simple osteogenic cultures toward constructs that reproduce selected features of bone or cartilage development [12]. Such models may be generated from embryonic stem cells, induced pluripotent stem cells (iPSCs), mesenchymal stromal/stem cells (MSCs), periosteal cells or combinations of skeletal and supportive lineages. Hydrogels and related matrices can provide biochemical ligands, hydration and mechanical support while remaining permissive to cell-mediated remodeling [[12], [13], [14]]. Bioprinting adds a further level of control by positioning osteogenic cells adjacent to endothelial, neural or stromal compartments and by introducing channels that can subsequently be perfused [15,16]. Accordingly, this review does not treat every mineralized three-dimensional culture as an organoid; instead, it examines the biological and engineering features that make a construct bone-like, how these features can be integrated and where the resulting models are most informative.

Spheroids, engineered bone-like tissues and organoids should therefore be distinguished. Spheroids are useful modular building blocks and may enhance osteogenic differentiation, but they often lack tissue-level organization [17,18]. Engineered constructs can reproduce geometry or mechanical behavior, yet may remain dominated by an exogenous scaffold [[19], [20], [21], [22]]. The term “bone organoid” is most informative when a model combines self-organized biological architecture with demonstrable skeletal function. This distinction is not intended as a rigid hierarchy; rather, it aligns terminology with the evidence presented for each model.

This review uses a structured narrative evidence-mapping approach rather than a formal systematic-review protocol. Representative studies were identified from the bone-organoid literature cited in this article and through supplementary searches of Crossref, PubMed/PMC and Europe PMC through July 2026 using combinations of “bone organoid,” “woven bone organoid,” “trabecular bone organoid,” “periosteum organoid” and “bone-organoid bioprinting.” Original experimental studies were eligible when they generated a three-dimensional skeletal construct and reported cell-driven organization, bone-specific matrix formation, remodeling, vascularization, mechanical behavior or application-specific validation.

Reviews, acellular scaffolds and generic bone tissue-engineering studies without evidence of organoid-like maturation were excluded from the study-level evidence map. Boundary cases—osteogenic spheroids and scaffold-dominated skeletal constructs—were retained deliberately so that the operational classification proposed in Section 3 could be applied rather than assumed. The resulting comparison is representative, not exhaustive, and prioritizes studies that clarify conceptual boundaries or provide direct evidence for maturation, multilineage interaction, biofabrication or translation.

Schematic overview of the development of organoid research and current bone-organoid engineering approaches. The timeline highlights major milestones from early tissue reaggregation and embryonic stem-cell isolation to intestinal organoids and the emergence of bone organoids. Bone organoids can integrate stem-cell sources (ESCs, MSCs and iPSCs), bioinks, hydrogels, extracellular matrix (ECM), growth factors and computer-aided design (CAD)-guided spatial control. Current strategies include scaffold-dependent fabrication, scaffold-free self-assembly, hybrid biofabrication and organoid-on-chip platforms, each with distinct strengths and limitations in structural support, cellular organization and physiological complexity for modeling bone development, disease and regeneration. The historical milestones were selected based on their transformative impact on organoid technology—from the demonstration of cellular self-organization (early 20th century), to the establishment of renewable pluripotent cell sources (1981), to the first defined-niche organoid system (2009), and finally to the emergence of bone-specific organoid engineering. The current strategies depicted (scaffold-dependent, scaffold-free, hybrid and organoid-on-chip) are compared using consistent criteria: structural support (the degree of exogenous material contribution), cellular organization (the extent of cell-driven vs. engineer-driven patterning), physiological complexity (the number of integrated lineages and functional readouts), and scalability (the feasibility of standardized production). These criteria are not intended as rigid rankings but as analytical dimensions to guide strategy selection based on specific research or translational objectives.

## 2 Biological mechanisms of bone regeneration

Bone repair is a staged process in which inflammatory signaling, progenitor-cell recruitment, matrix deposition, vascular invasion, mineralization and remodeling occur in a coordinated sequence [23]. Autologous bone graft remains the clinical reference standard because it provides viable osteogenic cells, an osteoconductive matrix and endogenous osteoinductive signals [24,25]. Its use is nevertheless constrained by donor-site morbidity, limited tissue volume, variable graft quality and additional operative time [26]. These limitations have sustained interest in bone tissue engineering and, more recently, in organoid-based strategies that aim to reconstruct the biological organization of a healing niche rather than provide a passive scaffold alone [27]. A key implication is that regenerative success cannot be assessed solely by the quantity of newly formed mineral. The timing and spatial distribution of tissue formation, vascular supply, remodeling capacity and mechanical competence are equally important.

Modern bone tissue engineering therefore combines cells, instructive materials, soluble factors and mechanical conditions in a deliberately staged manner. Bone marrow-derived MSCs (BMSCs) are widely used because they generate osteogenic and chondrogenic progeny and modulate neighboring cells through paracrine signaling [28] (Fig. 2). BMSCs alone, however, do not reproduce the complete repair response. Endothelial cells regulate perfusion and angiocrine signaling [29,30]; monocyte-lineage cells provide osteoclasts and inflammatory mediators [31,32]; neural elements influence vascular tone and skeletal-cell function [[33], [34], [35]]; and matrix-producing cells continuously reshape the physical niche. Experience from multicellular organoid systems in other tissues further indicates that incorporating relevant supporting lineages from the outset can improve maturation [36]. The central design challenge is therefore to organize these populations in space and time while avoiding unnecessary complexity.

**Fig. 2:**
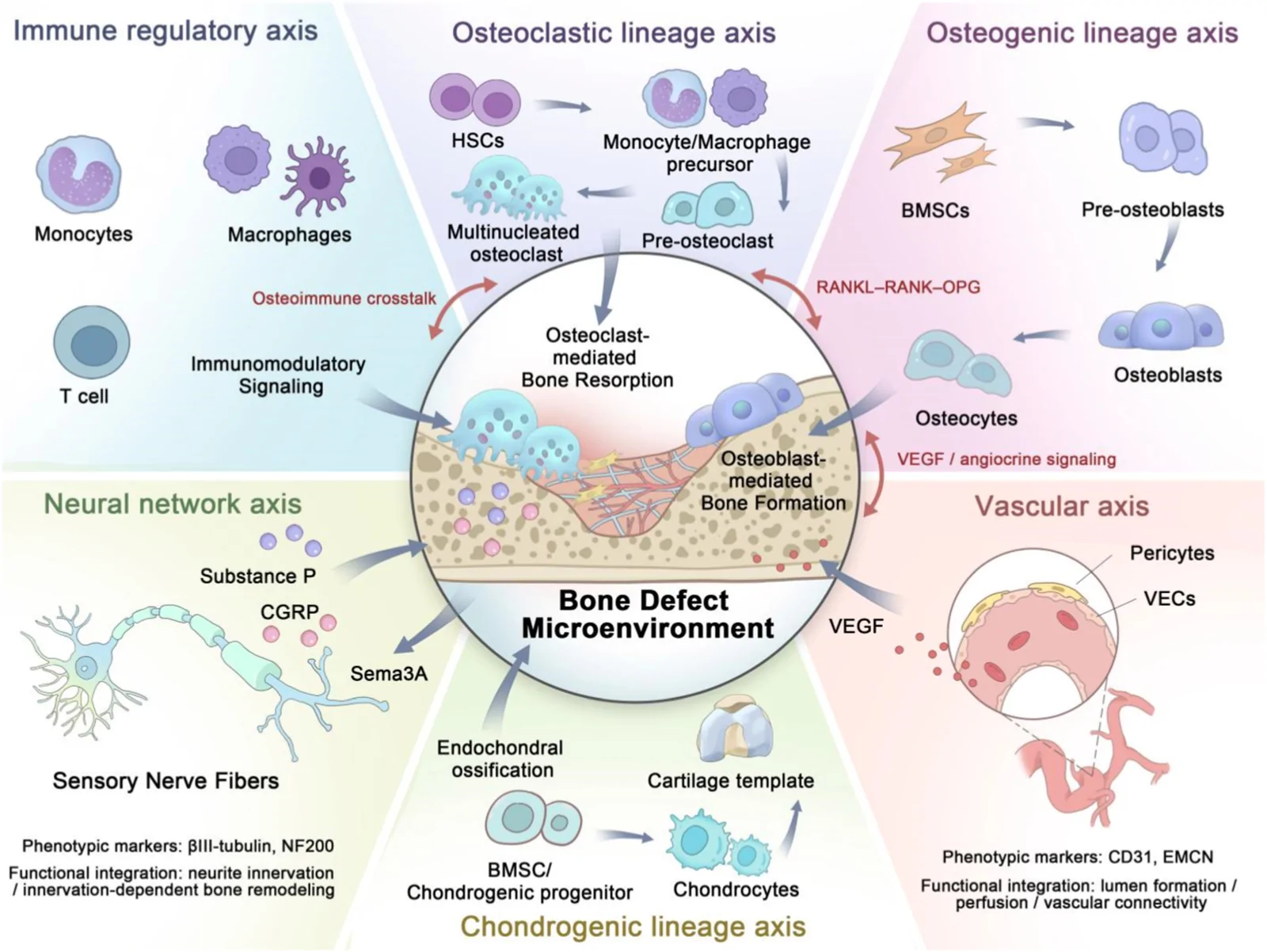
Integrated osteogenic-vascular-neural crosstalk in the bone-regenerative microenvironment.

The schematic integrates six interacting compartments—osteogenic, osteoclastic, chondrogenic, vascular, neural and immune—around the bone-defect microenvironment. Osteogenic and osteoclastic lineages are linked through formation-resorption coupling, including the RANKL–RANK–OPG axis; chondrogenic progenitors provide a developmental template for endochondral ossification; and immune cells regulate the inflammatory-to-reparative transition. Vascular and neural modules distinguish phenotypic markers from functional integration: vascular fidelity requires lumen formation, perfusion or vascular connectivity, whereas neural fidelity requires neurite innervation and, ideally, innervation-dependent regulation of bone remodeling. Together, these reciprocal interactions emphasize that multilineage marker expression alone is insufficient; functional integration should be demonstrated with lineage-appropriate readouts.

### 2.1 Anabolic–catabolic equilibrium: osteoblasts, osteocytes, and osteoclasts

Osteoblasts arise from committed mesenchymal progenitors and produce osteoid, an initially unmineralized matrix composed predominantly of type I collagen. Ascorbate supports collagen hydroxylation and matrix assembly, whereas subsequent deposition and growth of hydroxyapatite crystals convert the osteoid into mineralized tissue [37,38]. Sequential markers of osteoblast maturation include alkaline phosphatase, RUNX2, osterix, type I collagen, osteopontin, bone sialoprotein and osteocalcin [39]. These markers should be interpreted alongside matrix organization and mineral distribution, because expression of a late osteogenic marker alone does not establish the formation of functional bone-like tissue. Osteoblast-lineage cells also regulate the local remodeling environment through RANKL, osteoprotegerin and matrix-bound signals; their role in an organoid is therefore both constructive and regulatory [40].

Bone formation does not occur in isolation but is coupled to resorption. Osteoclasts are multinucleated cells formed by fusion of monocyte-macrophage precursors [41]. They adhere to mineralized matrix, acidify the resorption compartment and release proteolytic enzymes that remove mineral and collagen [42]. Resorption eliminates damaged tissue, creates space for new bone and releases or activates matrix-associated factors, including TGF-β- and BMP-related signals. Osteoclasts and their precursors also communicate directly with osteoblast-lineage cells, thereby coupling resorption to subsequent formation [43]. Incorporating osteoclasts can increase the physiological relevance of organoids designed to model osteoporosis, implant-associated osteolysis, tumor-induced bone destruction or antiresorptive therapy [44]. The practical challenge is to maintain quantifiable resorption without destabilizing the construct.

A subset of osteoblasts becomes embedded within the matrix it produces and differentiates into osteocytes. Through dendritic processes extending into canalicular spaces, osteocytes act as long-lived sensors of strain, fluid flow and microdamage [45,46]. They regulate osteoblast and osteoclast activity through mediators such as sclerostin, RANKL and osteoprotegerin. Reproducing a mature lacuno-canalicular network in vitro remains difficult; nevertheless, partial acquisition of an osteocyte-like phenotype may provide a more informative measure of mechanosensitivity than osteoblast markers alone [47].

### 2.2 The developmental template: chondrocytes and endochondral ossification

Mechanically unstable fractures commonly heal through indirect fracture healing involving endochondral ossification, whereas rigidly stabilized fractures can heal by direct intramembranous ossification [48]. Mesenchymal progenitors first generate a cartilage-rich callus that stabilizes the defect and tolerates relative hypoxia [49]. Chondrocytes then mature and hypertrophy, the matrix calcifies and vascular invasion introduces osteoprogenitors and remodeling cells [49]. Hypertrophic chondrocytes may undergo apoptosis, persist transiently or contribute to osteoblast-lineage populations, while calcified cartilage is progressively replaced by woven and subsequently lamellar bone [50]. This developmental sequence is attractive for organoid engineering because an initially avascular cartilage template can be generated in vitro and may retain endogenous cues that promote ossification after implantation. The outcome, however, depends strongly on template maturity: incomplete hypertrophy may delay ossification, whereas excessive calcification or premature cell death may impair host integration.

### 2.3 Bone–vascular–neural integration

Functional bone regeneration depends on reciprocal signaling among skeletal, vascular and neural compartments. These interactions are not secondary refinements; they determine whether newly formed tissue survives, remodels, senses load and integrates with the host.

Endothelial cells provide more than oxygen and nutrient delivery. Organ-specific signaling programs regulate endothelial barrier function, metabolism and paracrine output [51]. In bone, specialized microvessels and endothelial-derived signals influence osteoprogenitor maintenance, osteoblast differentiation and remodeling, while osteogenic cells secrete VEGF and other mediators that recruit and pattern the vasculature (Fig. 2) [52]. Vascularization can be promoted through endothelial co-culture, preformed capillary networks, sacrificial channels, perfusion or implantation into a highly vascularized host site.

**Table:** 

Strategy	Mechanism	Advantages	Limitations	Maturity	Best suited for
Endothelial co-culture	Direct seeding of ECs with osteogenic cells	Simple, reproducible, supports paracrine signaling	Limited vessel maturation, lack of perfusion	High (widely used)	In vitro differentiation studies
Preformed capillary networks	ECs self-organize into capillary-like networks	More physiological microvasculature	Fragile, difficult to perfuse, limited scale	Medium	Small-scale organoid models
Sacrificial channels	Fugitive inks create perfusable lumens	Enables immediate perfusion, large vessels	Lacks capillary resolution, may require endothelialization	Medium-High	Bulk transport in large constructs
Microfluidic perfusion	Continuous flow through integrated channels	Controlled shear, real-time sampling, oxygen delivery	Complex fabrication, limited self-organization	Medium	Organoid-on-chip platforms
In vivo vascularization	Implantation into vascularized host site	Native vascular integration, highest fidelity	Lacks in vitro control, host-dependent	High (in vivo)	Regenerative grafts

The choice among these strategies should be guided by the specific application: in vitro disease modeling may prioritize endothelial co-culture or preformed capillary networks for mechanistic studies; drug screening platforms benefit from microfluidic perfusion for controlled compound delivery; and regenerative grafts require in vivo vascularization or pre-vascularized constructs for host integration. Emerging hybrid approaches—such as combining sacrificial channels with endothelial sprouting—are beginning to bridge the gap between bulk perfusion and capillary-scale microvascularization.

These strategies are complementary: perfusable channels improve bulk transport, whereas capillary-scale networks reduce the diffusion distance between individual cells and the circulation.

Bone is richly supplied by sensory and sympathetic nerves. Mediators released by neural cells and peripheral nerves—including calcitonin gene-related peptide (CGRP), substance P, norepinephrine and neurotrophic factors—act on osteoblasts, osteoclasts, endothelial cells and immune cells [[53], [54], [55]]. Axonal guidance pathways, including Sema3A-related signaling, also intersect with bone-mass regulation and sensory innervation (Fig. 2) [56,57]. Neural integration is therefore relevant not only to pain modeling but also to tissue maturation and vascular regulation. Stable co-culture remains challenging because skeletal and neural cells often require different media, matrices and maturation schedules; consequently, most current bone organoids reproduce selected neurogenic signals rather than a fully organized sensory network. Several emerging strategies are being developed to address these co-culture challenges. Compartmentalized co-culture systems—such as microfluidic devices with physically separated but chemically connected chambers—allow skeletal and neural cells to be cultured in their respective optimal media while permitting paracrine signaling through controlled diffusion [58]. Sequential media switching may provide another practical approach: skeletal cells are first matured in osteogenic medium, followed by introduction of neural cells and gradual transition to a compromise medium that supports both populations without compromising skeletal matrix integrity. Alternatively, neural-derived factors may also provide cell-free cues for osteogenic and neurovascular regulation; for example, CGRP and substance P have been shown to promote osteogenic and/or angiogenic responses in bone-related experimental models [59]. More recently, neurotrophic spheroids have been incorporated into engineered ossification center-like organoids as modular components to provide local pro-regenerative cues [60]. These strategies collectively suggest that faithful neural integration may be achievable through a combination of spatial compartmentalization, temporal sequencing and factor-based signaling, rather than requiring a single unified culture condition.

### 2.4 Immune regulation of the regenerative niche

Bone repair begins with inflammation, and the quality and timing of this early response influence subsequent vascularization and osteogenesis [61,62]. Neutrophils and macrophages clear debris and release cytokines that recruit progenitors. Macrophage states are dynamic and cannot be adequately represented by a simple M1/M2 binary; inflammatory and reparative functions may be required at different stages [63]. Computational and AI-assisted approaches may complement experimental organoids by integrating time-dependent immune and stromal states and by supporting the design of microenvironment-targeted regenerative strategies [64]. Incorporating macrophages or defined immune-derived signals can therefore strengthen models of infection, implant response, inflammatory bone loss and fracture healing, provided that the culture system permits temporal state transitions rather than locking immune cells into a single phenotype [65].

## 3 Redefining the bone organoid: challenges and a practical framework

Despite rapid progress, no consensus definition of a bone organoid has been established. This ambiguity reflects both the biological complexity of bone and wide variation in how three-dimensional skeletal constructs are produced and evaluated. Native bone is not merely a mineralized cell mass; it is a hierarchically organized, continuously remodeled tissue in which extracellular matrix deposition, mineralization, cellular homeostasis and mechanical adaptation are tightly coupled. The term “bone organoid” should therefore not be applied indiscriminately to all three-dimensional osteogenic aggregates.

A practical definition should prioritize the bone-specific properties reproduced by a construct while recognizing that engineered guidance can provide an initial boundary or transient support. Guidance alone, however, does not establish organoid identity. In the framework proposed here, a high-fidelity bone organoid is evaluated across three interdependent domains: structural mimicry, functional fidelity and biomechanical integrity (Fig. 3) (see Fig. 4).

**Fig. 3:**
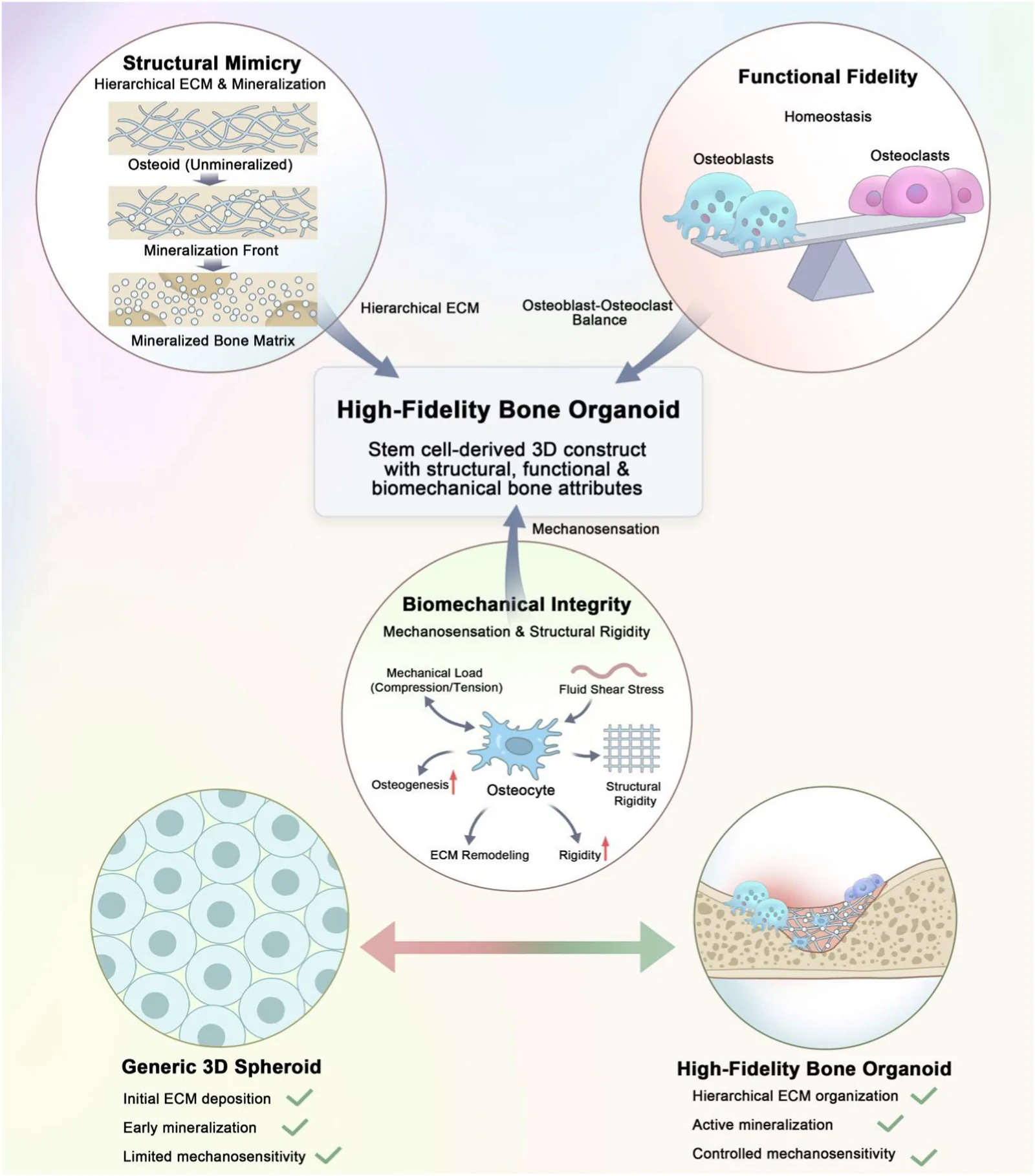
Characterization of a high-fidelity bone organoid.

**Fig. 4:**
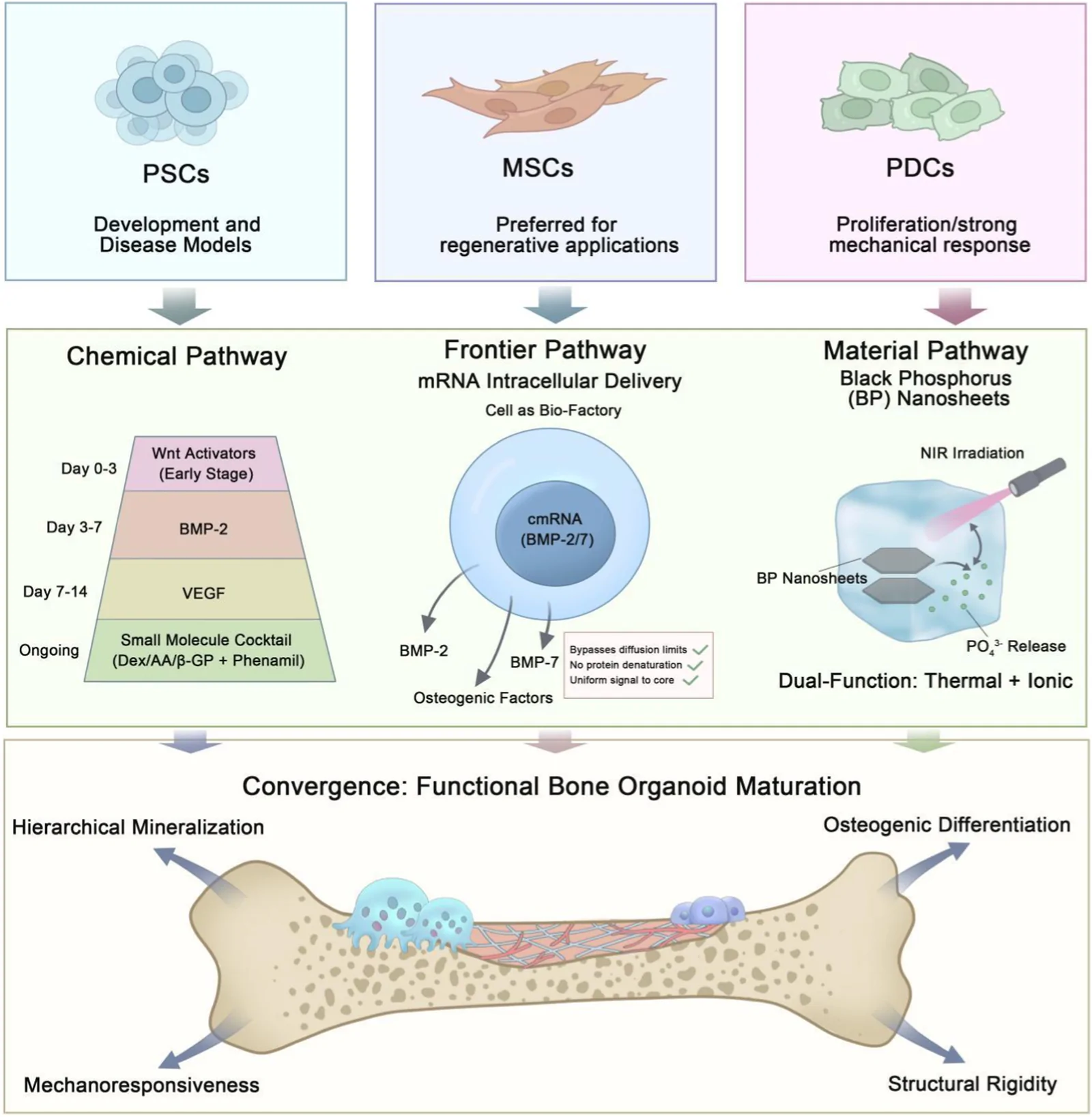
**Cellular sources and regulatory strategies for functional bone-organoid maturation.** Pluripotent stem cells (PSCs) are particularly suited to developmental and disease modeling, mesenchymal stromal/stem cells (MSCs) to regenerative applications and periosteum-derived cells (PDCs) to mechanically responsive bone repair. Maturation can be regulated through staged chemical induction with Wnt modulators, BMP-2, VEGF and small-molecule cocktails; intracellular delivery of chemically modified mRNA encoding factors such as BMP-2 or BMP-7; and black-phosphorus nanosheets that provide photothermal stimulation and phosphate-ion release. These strategies converge on hierarchical mineralization, osteogenic differentiation, mechanosensitivity and structural competence.

### 3.1 Structural mimicry: hierarchical extracellular matrix organization and mineralization

Structural mimicry refers to the capacity of a bone organoid to reproduce the hierarchical organization and progressive mineralization of native bone ECM. During physiological bone formation, osteogenic cells first deposit collagen-rich, unmineralized osteoid. Mineralization then advances from discrete fronts, gradually transforming the osteoid into a consolidated mineralized matrix.

A high-fidelity bone organoid should reproduce this sequence rather than merely accumulate isolated calcium deposits. Evidence should include spatially organized ECM deposition, a recognizable transition from unmineralized osteoid to actively mineralizing regions and eventual formation of a consolidated mineralized matrix [66,67]. These features distinguish tissue-specific bone-like organization from a generic three-dimensional spheroid.

### 3.2 Functional fidelity: coupled formation, resorption and bone homeostasis

Functional fidelity is the ability of a construct to reproduce the coordinated cellular activities that maintain bone homeostasis. Native bone is sustained by coupling between osteoblast-mediated matrix formation and osteoclast-mediated resorption. Osteoblasts produce osteoid and promote mineralization, whereas osteoclasts remove aged or damaged matrix and release signals that regulate subsequent bone formation [32,68].

A high-fidelity bone organoid should therefore not be defined by osteogenic-marker expression or mineral deposition alone. Ideally, it should display coordinated matrix formation, resorption and remodeling, together with functional communication among osteoblast-lineage cells, osteoclast-lineage cells and, where relevant, osteocytes. Demonstrating coupled anabolic and catabolic activity provides a more physiologically meaningful measure of bone-like homeostasis and broadens the model's utility for studying skeletal remodeling.

### 3.3 Biomechanical integrity: mechanosensation and structural rigidity

Biomechanical integrity is a third defining attribute of a high-fidelity bone organoid. Native bone is both load-bearing and highly mechanosensitive; its cells continuously detect changes in mechanical loading, fluid flow and matrix stiffness and convert these physical cues into biological responses.

Relevant mechanical cues include compression, tension, fluid shear stress and changes in ECM stiffness. Osteocytes and osteogenic cells sense these stimuli and regulate osteogenesis, matrix remodeling and mineral deposition. As mineralization increases local stiffness, the evolving matrix further alters cellular mechanosensation, creating a reciprocal relationship between tissue maturation and mechanical function.

A bone organoid need not reproduce the full strength of native bone. It should, however, possess sufficient structural coherence to maintain its three-dimensional architecture and exhibit measurable responses to mechanical stimulation. Evidence of mechanosensation, ECM remodeling and progressive mechanical competence helps distinguish a functional bone organoid from a passive cell aggregate.

On the basis of these criteria, a bone organoid is a viable cell-derived three-dimensional tissue in which endogenous cell-cell and cell-matrix interactions generate bone-specific organization and function beyond the geometry imposed by culture or fabrication. Engineered guidance may initiate or stabilize the construct, but cell-produced ECM, biologically regulated active mineralization that can be distinguished from material-derived mineral signals, and at least one dynamic skeletal function must emerge during maturation. A high-fidelity bone organoid additionally demonstrates hierarchical matrix maturation, coupled formation and resorption, and a controlled response to mechanical perturbation.

This framework distinguishes evidence classes without assuming that every construct must progress along a single path. Spheroids can be useful modular building blocks but do not qualify as organoids without bone-specific tissue organization. Bone-like microtissues demonstrate cell-derived osteoid or controlled mineralization but lack complete hierarchy or dynamic function. Engineered skeletal constructs can reproduce geometry, perfusion or remodeling with high precision while remaining dominated by exogenous material architecture. Bone organoid and high-fidelity labels should therefore be assigned from the evidence reported, as summarized in Table 1, rather than from three-dimensional morphology or author terminology alone.

**Table 1:** Operational classification of three-dimensional skeletal models.

Operational class	Required biological evidence	Role of engineered material	Distinguishing boundary
Osteogenic spheroid	Self-assembled aggregate with viable cell-cell contact; osteogenic markers or mineral staining may be present	Absent or minimal; material-free status alone does not establish an organoid	Lack of organized bone ECM and dynamic function
Bone-like microtissue	Cell-derived collagen/osteoid and longitudinal, material-controlled evidence of de novo mineralization	A temporary mold or permissive matrix may support formation but should not supply the principal mineral signal	Incomplete hierarchy, remodeling and adaptive function
Engineered skeletal construct	One or more bone-relevant structures or functions reproduced under externally imposed architecture	Persistent scaffold, demineralized matrix, CAD geometry or material mechanics dominate organization	Scaffold-dominated architecture without intrinsic organoid identity
Bone organoid	All core criteria: emergent organization, cell-derived bone ECM, controlled active mineralization and at least one dynamic skeletal function	Guidance is permissible when transient or demonstrably permissive to endogenous organization and remodeling	Core organoid identity with application-dependent validation
High-fidelity bone organoid	Bone-organoid core criteria plus hierarchical ECM/mineral maturation, coupled formation–resorption, and controlled mechanosensitivity/mechanical competence	Acellular baselines and scale-resolved mechanics must separate scaffold contribution from tissue maturation	Multidomain fidelity; lineage integration is application dependent

A functional bone organoid is expected to reproduce three interdependent features of native bone: structural mimicry, including hierarchical extracellular matrix (ECM) organization and progressive mineralization; functional fidelity, including coupled bone formation and resorption; and biomechanical integrity, including mechanosensation and adaptive responses to loading. These properties distinguish high-fidelity bone organoids from conventional three-dimensional spheroids. It is important to acknowledge, however, that certain osteogenic spheroids can deposit endogenous ECM, mineralize and respond to mechanical stimulation. The distinction is therefore not categorical but graded: spheroids occupy a lower tier of organizational complexity, while high-fidelity bone organoids represent a higher tier characterized by hierarchical matrix architecture, spatiotemporally regulated mineralization and coupled anabolic-catabolic activity. This tiered classification, aligned with the framework proposed in Section 3, emphasizes that classification should be based on bone-specific functional evidence rather than three-dimensional morphology alone.

### 3.4 Operational classification and material-aware evidence thresholds

The five labels in Table 1 are operational classes rather than a simple maturity ladder. In particular, an engineered skeletal construct can be sophisticated and clinically useful while remaining scaffold-dominated; its placement outside the organoid class is a statement about the source of organization, not about quality. Engineered guidance may define an initial boundary, channel or temporary support, but organoid status requires bone-specific architecture and function to emerge through cell-cell and cell-matrix interactions beyond the imposed geometry.

Core bone-organoid criteria are: (i) a viable cell-derived three-dimensional tissue that develops organization beyond simple aggregation; (ii) cell-produced collagenous or osteoid-like ECM with spatially and temporally resolved mineralization; (iii) evidence that new mineral is biologically deposited rather than inherited from the material; and (iv) at least one dynamic skeletal function, such as regulated matrix formation, formation-resorption coupling, a functional vascular niche or a controlled mechanobiological response. Vascular, neural, immune and marrow components are advanced, application-dependent features rather than universal core requirements. A high-fidelity designation requires the core criteria plus evidence across all three domains in Fig. 3: hierarchical matrix maturation, coupled formation and resorption, and controlled mechanosensitivity with mechanical competence attributable to the developing tissue.

## 4 Construction elements: cellular candidates and induction strategies

Bone-organoid construction depends on the compatibility of the cell source, induction sequence, material environment and intended endpoint. No single design is optimal for developmental modeling, drug screening and regenerative implantation. Rational design should begin with the biological question and use the minimum complexity required to answer it.

### 4.1 Cellular candidates: matching cell source to purpose

Cell source influences differentiation potential, maturation time, donor variability, immunogenicity and scalability. Pluripotent stem cells are particularly suited to developmental and genetic studies, adult stromal cells to repair-oriented models and tissue-specific progenitors to questions centered on a defined skeletal niche. Selection should therefore be driven by function rather than availability alone.

Embryonic stem cells and iPSCs are especially valuable for modeling early human skeletogenesis and genetic skeletal disorders [69,70]. Their broad developmental potential permits stepwise induction through mesodermal, paraxial mesodermal, sclerotomal, chondrogenic and osteogenic states, enabling study of transitions that cannot be captured with adult primary cells. Patient-specific iPSCs retain the relevant genetic background and can be paired with gene-corrected isogenic controls, a major advantage for precision disease modeling [71]. Limitations include lengthy differentiation protocols, line-to-line variability, residual immature cells and the need to verify lineage purity before assembling a multicellular organoid.

MSCs, particularly BMSCs, remain the most practical cell source for adult bone-regeneration models [72]. They are comparatively accessible, expandable, responsive to established osteogenic and chondrogenic protocols and readily incorporated into spheroids or printable hydrogels. Their contribution extends beyond differentiation: MSCs release immunomodulatory, angiogenic and trophic factors that influence macrophage behavior, endothelial organization and host integration [73]. However, donor age, tissue origin, passage number, culture density and preconditioning can substantially alter phenotype. These variables should be reported to enable meaningful comparison across studies.

The periosteum contains skeletal progenitors that contribute directly to fracture repair and exhibit both osteogenic and chondrogenic potential [74]. Because periosteum-derived cells originate from a mechanically active, highly vascularized niche, they may be particularly informative for modeling cortical repair, periosteal callus formation and load-responsive osteogenesis. Their broader use is limited by tissue availability and heterogeneous isolation methods; accordingly, they are most valuable when periosteal identity is central to the biological question.

Endothelial cells, pericytes, monocyte-lineage cells, neural cells and marrow stromal populations can be incorporated when their function is essential to the model. Each added lineage, however, introduces additional variability. Cell ratio, order of seeding, direct versus indirect contact and medium compatibility all influence outcome. In many settings, sequential assembly—first establishing a stable osteogenic or chondrogenic unit and then adding vascular, immune or neural components—may be more reproducible than mixing all cell types at the outset.

The cellular sources (PSCs, MSCs, PDCs), molecular induction strategies (Wnt modulators, BMP-2, VEGF, small molecules), genetic delivery approaches (modified mRNA encoding BMP-2/BMP-7) and material-based regulation (black-phosphorus nanosheets) depicted in this figure are independent and potentially combinable design variables, not fixed associations. For example, MSCs can be combined with modified mRNA delivery, PSCs can be modulated by small-molecule cocktails, and any cell source can be cultured with black-phosphorus-containing matrices. The figure illustrates representative strategies rather than exclusive pairings, and researchers should select and combine these variables based on the specific biological question, intended application and desired maturation timeline.

### 4.2 Induction systems: driving functional maturation

Induction protocols govern not only lineage commitment but also the timing of proliferation, matrix production, vascular recruitment and mineralization. Excessive osteogenic stimulation at an early stage may impair endothelial survival or restrict expansion, whereas delayed mineralization may leave the construct mechanically weak. Staged protocols are therefore generally more informative than continuous exposure of all cell types to a single medium.

Continuous exposure to a single factor is unlikely to reproduce developmental progression; signal identity, sequence, dose and duration are all important. Wnt modulation can promote mesodermal specification, whereas TGF-β and BMP pathways support chondrogenic or osteogenic commitment in a context-dependent manner [75], and VEGF promotes vascular recruitment and osteogenic-vascular coupling. Co-delivery of BMP-2- and VEGF-A-encoding modified mRNAs illustrates how osteogenic and angiogenic signals can be coordinated within a scaffold (Fig. 5A) [76]. Sequential induction can separate early expansion, intermediate lineage commitment and later matrix maturation, thereby reducing antagonistic effects between cell populations (see Fig. 6).

**Fig. 5:**
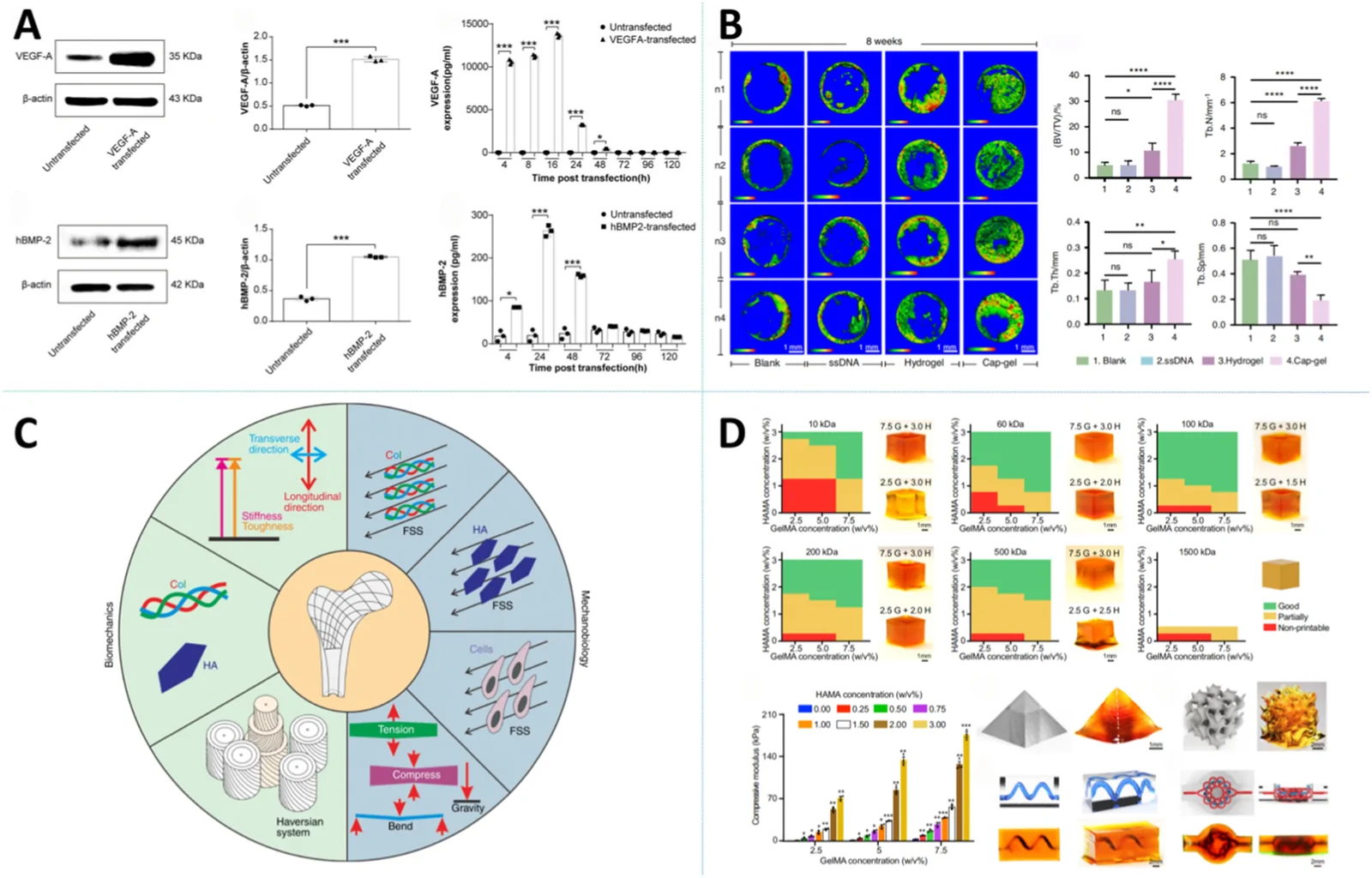
Representative molecular, material, mechanical and regenerative cues for bone-organoid engineering (A) Transient bone morphogenetic protein 2 (BMP-2) and vascular endothelial growth factor A (VEGF-A) expression after modified-mRNA transfection, illustrating local, short-duration osteogenic and angiogenic signaling [76]. (B) Micro-computed tomography (micro-CT) reconstruction and bone morphometric analysis of rat cranial defects treated with mineralized DNA-tetrahedron-structured hydrogels after 8 weeks [77]. (C) Biomechanical and mechanobiological cues in bone matrix, including collagen, hydroxyapatite, cells, fluid shear stress, stiffness, compression, tension and bending [78]. (D) Printability, mechanical properties and digital light processing (DLP) -fabricated structures produced with gelatin methacryloyl (GelMA)/hyaluronic acid methacrylate (HAMA) bioinks, illustrating the influence of bioink composition on fabrication fidelity and construct stiffness [79].

**Fig. 6:**
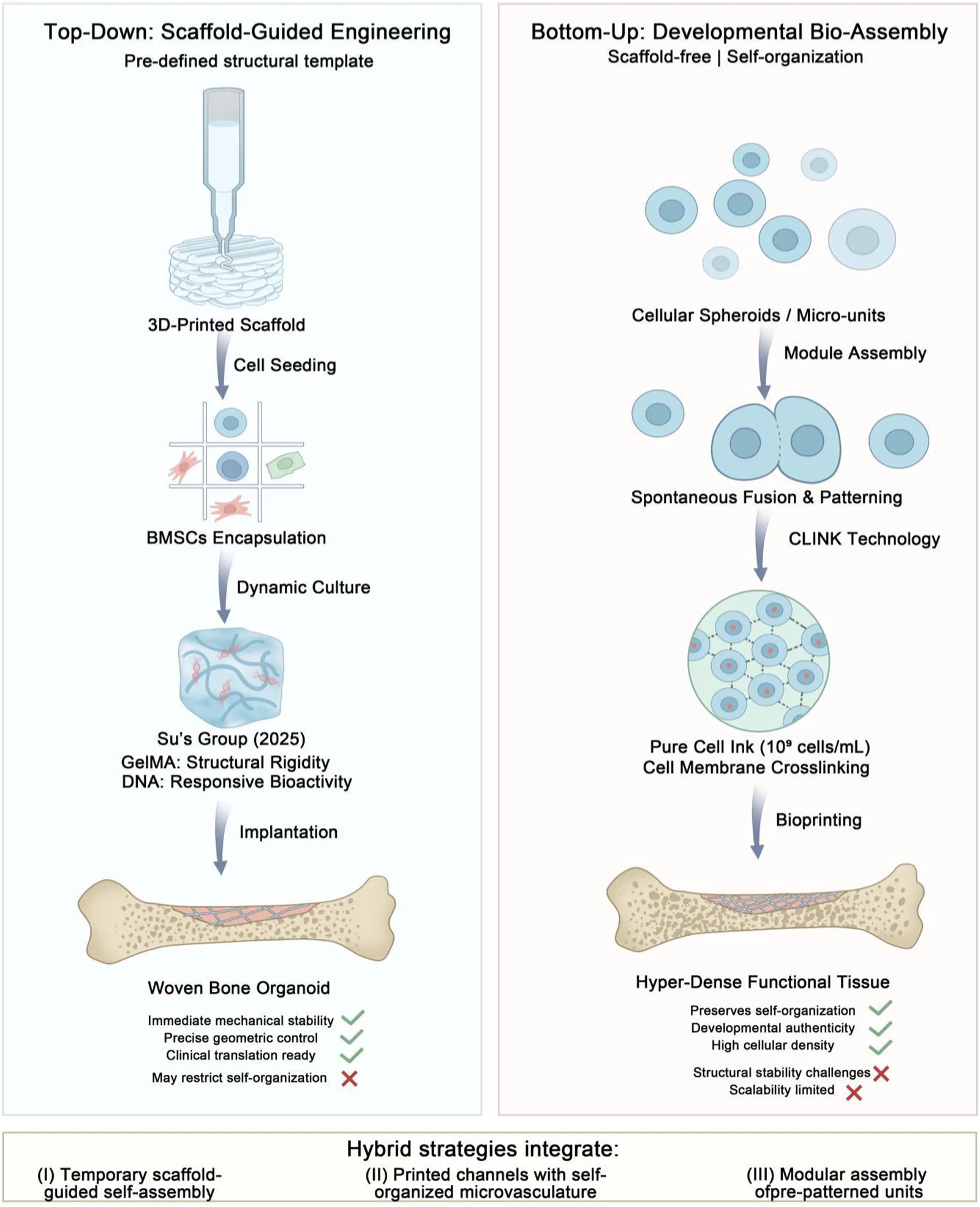
Top-down and bottom-up strategies for bone-organoid engineering (A) Top-down engineering uses predefined three-dimensional scaffolds to guide BMSC encapsulation, spatial organization and dynamic culture. GelMA/DNA networks provide initial structural stability and responsive bioactivity, although persistent scaffolds may restrict self-organization. (B) Bottom-up engineering assembles spheroids or cellular micro-units through fusion and patterning. CLINK denotes a cell-surface photocrosslinking strategy for cell-dense, low-biomaterial bioinks; it can preserve developmental self-organization, but structural stability and scalability remain limiting. Hybrid strategies may combine structural fidelity with biological potency.

Small molecules are often less expensive, more batch-consistent and easier to control temporally than recombinant proteins [80]. The conventional osteogenic combination of dexamethasone, ascorbate and β-glycerophosphate remains useful as a baseline but does not reproduce a complete developmental program. Additional modulators of Wnt, BMP, hedgehog, retinoic acid or epigenetic pathways can be selected to address specific maturation bottlenecks. Phenamil, for example, has been investigated as an enhancer of BMP-associated osteogenic signaling [81]. Because small molecules may act on multiple pathways, dose-response studies and defined washout periods are needed to distinguish developmental effects from non-specific stress or accelerated mineral precipitation.

Inorganic and two-dimensional nanomaterials can provide structural and biochemical cues [82]. Nano-hydroxyapatite resembles the mineral phase of bone and can enhance protein adsorption, cell attachment and local calcium-phosphate nucleation [83]. A mineralized DNA-tetrahedron-structured hydrogel has also been reported to combine immunomodulatory and osteogenic signals and to improve micro-CT indices of rat cranial bone regeneration at 8 weeks (Fig. 5B) [77]. Black-phosphorus-based materials can incorporate photothermal or drug-delivery functions and release phosphate-containing degradation products [84]. These potential benefits must be balanced against aggregation, non-uniform distribution, light-dependent toxicity and changes in hydrogel rheology. Material evaluation should therefore include cell survival, spatial mineralization and imaging compatibility in addition to bulk osteogenic readouts.

Chemically modified mRNA enables transient production of proteins such as BMP-2, BMP-7, VEGF and neurotrophic factors without genomic integration. Cells within the construct can thereby serve as short-lived local sources of a desired signal, reducing the need for repeated administration of unstable recombinant proteins [[85], [86], [87]]. Performance depends on carrier composition, transfection efficiency, innate immune activation, intracellular persistence and the spatial range of the encoded protein. Delivery to a dense organoid core remains difficult because ECM can impede nanoparticles and peripheral cells may dominate uptake. Pre-transfection, repeated low-dose exposure, microchannel delivery and cell-type-specific carriers may therefore be more reliable than a single bolus delivered after maturation.

### 4.3 Microenvironmental engineering: reproducing the skeletal niche

The native skeletal niche is spatially heterogeneous, dynamic and continuously remodeled. Engineered models must therefore account for mechanical stimuli, oxygen and nutrient transport, biochemical gradients and the changing requirements of cells during maturation.

Bone cells continuously sense matrix stiffness, fluid flow, deformation and surface geometry (Fig. 5C) [78]. These cues are transduced through integrins, focal adhesions, the cytoskeleton, ion channels and nuclear mechanosensors. RUNX2 activity is one of several downstream responses modulated by substrate mechanics and cell shape [88]. The stiffness of a bone organoid should not be regarded as a fixed material property: cell-mediated remodeling, collagen deposition and mineralization progressively alter the local modulus. Hydrogel viscoelasticity and stress-relaxation behavior, rather than static stiffness alone, can critically influence MSC osteogenic differentiation and matrix remodeling [89]. Dynamic loading, perfusion-induced shear or cyclic compression is best introduced after an initial consolidation period and evaluated against acellular and time-zero mechanical baselines.

Oxygen and nutrient diffusion become limiting as organoid diameter and matrix density increase. A viable peripheral shell may therefore coexist with a hypoxic or necrotic core even when average live/dead measurements appear acceptable. Microfluidic perfusion and bioprinted channels reduce transport distances and enable defined flow conditions [[90], [91], [92], [93]]. Mild hypoxia may support early chondrogenesis or angiogenic signaling, whereas sustained severe hypoxia compromises matrix maturation and cell survival. Spatial viability mapping, oxygen-sensitive probes, lactate measurements and section-based analyses are more informative than a single whole-construct readout.

Bone ECM stores and presents cytokines, growth factors, adhesion ligands and mineral ions in a spatiotemporally restricted manner [94]. Hydrogels such as GelMA can be engineered to retain proteins, bind heparin-affine factors or degrade at defined rates. This allows adjacent regions to present distinct cues—for example, a softer chondrogenic compartment next to a stiffer osteogenic compartment. The design challenge is to maintain a gradient long enough to influence cell fate while preserving cell migration and matrix remodeling. Direct measurement of factor distribution is preferable to assuming that a printed pattern remains stable throughout culture.

Bone-organoid studies frequently assess constructs at a single endpoint, although the same model may pass through proliferative, matrix-forming, mineralizing and remodeling phases. Longitudinal sampling is required to distinguish transient stress from accelerated maturation. Terminal histology and molecular analyses can be complemented by non-destructive measurements, including secreted markers, optical coherence imaging, appropriately dosed micro-computed tomography and mechanical testing of replicate constructs. When evaluating a new material or growth factor, time-resolved analysis is essential because an early increase in mineral signal may be transient or reflect non-specific precipitation.

## 5 Strategies for bone-organoid construction

Bone organoids can be constructed through top-down structural guidance, bottom-up self-organization or hybrid strategies. These approaches are complementary rather than mutually exclusive. In many systems, an engineered mold or transient matrix defines the initial geometry, whereas cell-cell interactions and endogenous ECM determine the mature tissue architecture.

### 5.1 Top-down strategies: scaffold- and geometry-guided engineering

Top-down strategies use a predefined scaffold, mold or channel network to control tissue formation and overall geometry [[95], [96], [97]]. They are particularly useful when pore size, external shape, load transfer or perfusion paths must be specified in advance. Ceramics, polymers, decellularized matrices and crosslinked hydrogels can provide structural templates [98]. Their main advantage is reproducible macroarchitecture; their principal limitation is that a persistent material may dominate cell behavior or restrict reorganization into a more physiological structure. An effective top-down bone-organoid system therefore requires sufficient early stability together with later permissiveness for cell-driven remodeling.

Dynamic hydrogels illustrate how top-down guidance can be combined with cell-mediated organization. A GelMA/DNA dual-network system developed by Zhu and colleagues provided initial mechanical support while retaining supramolecular interactions that could reorganize during culture [99]. BMSCs embedded in such networks can deposit endogenous matrix within a material that is more adaptable than a permanently rigid scaffold. These constructs may be suitable for regenerative implantation because they can be delivered in a defined form while continuing to evolve after placement. Their performance, however, should be separated into material-driven effects, cell-intrinsic effects and genuine organoid-level organization.

Additive manufacturing further extends top-down design by generating patient-specific external shapes and controlled internal pore networks [[100], [101], [102]]. Printed ceramic or polymer frameworks can preserve defect geometry, guide tissue ingrowth and provide routes for vascular invasion. In bone-organoid applications, these frameworks may be seeded with preformed spheroids, filled with cell-laden hydrogels or used as reusable molds for self-assembled tissue. Their clinical advantage is geometric compatibility with the defect; their biological drawback is that a high material fraction can reduce the cell-dense character of the organoid. Hybrid designs therefore increasingly minimize permanent scaffold volume while retaining the architecture required for handling and implantation.

### 5.2 Bottom-up strategies: developmental bioassembly and materials-assisted engineering

Bottom-up strategies begin with cells or small multicellular units and generate larger tissues through fusion, sorting, compaction and matrix deposition [103,104]. Spheroids are widely used because they promote cell-cell contact, retain endogenous ECM and can be produced at scale [105]. They should be viewed as modular building blocks rather than miniature end-stage tissues. Differential adhesion, local oxygen gradients, cortical tension and matrix remodeling govern whether adjacent units fuse uniformly or remain compartmentalized [106]. Bottom-up systems are biologically adaptable but difficult to standardize when spheroid size, maturation state or surface matrix varies across batches.

Developmental priming is a particularly important bottom-up strategy. MSC microtissues can first be directed toward a cartilage-like state and then assembled into a larger construct capable of endochondral ossification. Bioactive nanomaterials incorporated within individual spheroids can provide local mineral or signaling cues without a continuous external scaffold. Developmentally primed cartilage microtissues retain matrix-bound signals and cellular states that support vascular invasion and bone formation after implantation [107,108]. This approach resembles callus-mediated healing more closely than direct osteoblast seeding onto a rigid scaffold, but it requires prolonged pre-culture and may show heterogeneous hypertrophy, contraction or deformation during fusion. Materials-assisted variants have incorporated collagen and black phosphorus into MSC spheroids within biodegradable hydrogels, improving viability and osteogenic differentiation [109]. Other scaffold-free spheroids functionalized with black phosphorus-graphene oxide heteronanostructures have enabled co-delivery of stem cells and dexamethasone and promoted neovascularization and bone regeneration in vivo [110]. More recent designs combining ssDNA-functionalized carbon nanotubes, gelatin-bound black phosphorus nanosheets and dexamethasone have sought to improve oxygen transport while coordinating osteogenic induction and macrophage-mediated immunoregulation [111].

Cell-surface photocrosslinking strategies, referred to here as CLINK, can reduce bulk biomaterial by introducing photocrosslinkable groups at the cell surface, enabling highly cellular inks and complex geometries [112,113]. High cell density may enhance paracrine signaling and matrix production, but printing and crosslinking must preserve viability. Endothelial networks can reduce hypoxia-associated necrosis and support long-term maturation by facilitating perfusion and nutrient transport [114]. Cleavable or removable bioinks provide transient printability and then yield tissues less dominated by exogenous material (Fig. 5D) [79]. In bone organoids, programmed placement is therefore most informative when followed by demonstrated post-print self-organization: printing defines the initial architecture, whereas cells must generate the mature microstructure.

#### 5.2.1 Hybrid strategies: combining top-down guidance with bottom-up self-assembly

Top-down and bottom-up strategies are complementary rather than mutually exclusive, and their integration—termed hybrid biofabrication—offers a powerful paradigm for bone-organoid engineering. Hybrid strategies can be categorized into three main types based on the temporal and spatial relationship between engineered guidance and cellular self-organization.

Type I: Temporary scaffold-guided self-assembly. In this approach, a transient scaffold or mold provides initial structural support and defines macrogeometry, but progressively degrades or remodels to permit cell-driven reorganization [99,115]. The GelMA/DNA dual-network hydrogel system developed by Zhu and colleagues exemplifies this category: the hydrogel provides initial mechanical support through DNA crosslinking, while supramolecular interactions allow gradual network reorganization as BMSCs deposit endogenous ECM, ultimately yielding woven bone organoids via intramembranous ossification. Similarly, molecularly cleavable bioinks enable high-fidelity DLP printing followed by triggered degradation of the synthetic component, leaving behind a cell-dominated tissue with minimal exogenous material burden.

Type II: Printed channels with self-organized microvasculature. Here, bioprinting defines large-scale perfusable conduits, while cells spontaneously form capillary-scale networks in the intervening space. The Neo-Organoid Visualization and Assembly (NOVA) strategy achieves this by CAD-guided printing of tubular architectures followed by in situ unidirectional vascularization through endothelial cell self-organization. Sacrificial printing and endothelialized microchannel strategies can create immediately perfusable conduits, while endothelial cells in the surrounding hydrogel form capillary networks that sprout toward and anastomose with these channels [[116], [117], [118]].

Type III: Modular assembly of pre-patterned units. This strategy combines bottom-up spheroid/organoid units with top-down spatial patterning. Developmentally primed cartilage microtissues can be printed in defined spatial arrangements and subsequently undergo fusion and endochondral ossification, yielding organized bone tissue with region-specific maturation [119,120]. ECM-DNA-CPO-based bionic matrix hydrogels fabricated through photocrosslinking and dynamic self-assembly have enabled the sequential generation of mineralized bone organoids in vitro and vascularized bone-organoid tissues following in vivo maturation [121].

The central principle underlying all hybrid strategies is that engineered guidance should provide support precisely when and where needed—defining geometry, enabling surgical handling and establishing transport pathways—while remaining permissive to, or eventually being replaced by, cell-driven tissue organization.

### 5.3 Organoid-on-a-chip and hybrid perfusion strategies

Microfluidic and organ-on-a-chip platforms have increasingly been applied to reproduce selected structural and functional features of the skeletal microenvironment under dynamically controlled conditions [122,123]. Early vascularized bone-on-chip models incorporated hydroxyapatite into fibrin-based extracellular matrices and demonstrated the formation of three-dimensional microvascular networks, with vascular development evaluated by sprout length, sprouting rate, branch number and lumen diameter [122]. Perfusion-based osteogenesis-on-chip systems have further demonstrated that fluid flow can act as both a transport mechanism and a mechanical cue: Bahmaee et al. [123] tested flow rates of 0.8–3.2 mL/min, corresponding to approximately 0.2–1.4 Pa of shear stress, and showed that intermittent rather than continuous flow enhanced osteogenic differentiation and matrix deposition. More complex skeletal microphysiological systems have integrated mineralized endosteal-like compartments with three-dimensional vascular networks, enabling investigation of spatial interactions between bone-associated and perivascular niches [124,125]. Notably, recent microfluidic platforms have also enabled high-throughput generation of self-assembled, vascularized and mineralized three-dimensional bone organoids, providing more direct evidence for the convergence of bone-organoid and microfluidic technologies [126]. Collectively, these studies indicate that validation of bone-organoid-on-chip systems should extend beyond gross morphology to include defined perfusion conditions and shear stress, oxygen distribution, osteogenic and mineralization endpoints, vascular network formation and perfusability, endothelial barrier function, and, where relevant, mechanically induced responses.

Building on these advances, we envision that future bone-organoid-on-chip strategies could increasingly use biomimetic microarchitecture not only as a culture compartment but also as a spatial template for tissue self-organization. One promising future direction is to develop a trabecular bone-mimetic organ-on-a-chip platform in which three-dimensional BMSC spheroids are spatially assembled along trabecular-like channels while endothelial cells are introduced to generate vascular structures within the same predefined architecture, ultimately forming a vascularized bone organoid with trabecular-like structural organization. Rather than considering this configuration an established construction strategy, we propose it as a future direction in which geometric guidance and cellular self-organization are combined to bridge microscale vascularization with macroscale bone architecture. Its biological fidelity will require systematic optimization and validation of perfusion rate and shear stress, local oxygen gradients, endothelial barrier integrity and perfusability, mechanical stimulation, and osteogenic and vascular maturation. Such platforms may ultimately provide a controllable framework for investigating how bone-like geometry, vascular organization and mechanical cues collectively regulate bone-organoid maturation and function.

### 5.4 Study-level evidence map and classification

Table 2A, Table 2B, Table 2C, Table 2DA–2D apply the operational terminology to representative original studies. The companion-table format separates model design, biological organization, functional evidence and validation while retaining the same study set. Classification is based on the evidence reported, not on the label used by the original authors. Consequently, some scaffold-free constructs remain osteogenic spheroids or bone-like microtissues, whereas some highly functional remodeling systems are classified as engineered skeletal constructs because pre-existing material architecture dominates organization.

**Table 2A:** Representative studies: cell source, developmental route and construction.

Study	Cell source/species	Developmental route	Construction/matrix
Kérourédan et al., 2019 [127]	Endothelial cells + MSCs; mouse calvarial defect	Direct repair	In situ laser-assisted patterning in collagen/VEGF
Akiva et al., 2021 [128]	Human BMSCs	Intramembranous/woven bone	Three-dimensional culture with cell-produced collagen matrix
Park et al., 2021 [129]	Primary murine osteoblasts + bone-marrow mononuclear cells	Trabecular remodeling model	Demineralized bovine bone paper
Li et al., 2021 [109]	MSC spheroids; rodent validation	Direct osteogenic induction	Collagen/black-phosphorus spheroids in degradable hydrogel
Liu et al., 2022 [110]	MSC spheroids; rodent validation	Direct osteogenic induction	Scaffold-free spheroids coated with 2D heteronanostructures and dexamethasone
Li et al., 2023 [130]	Human BMSCs + human DPSCs	Endothelial priming followed by osteogenic induction	Temporary pNIPAAm mold removed after aggregation
Liu et al., 2024 [111]	MSC spheroids; rat calvarial defect	Direct osteogenic induction	ssDNA-carbon nanotube/black-phosphorus/dexamethasone heterostructure
Zhu et al., 2025 [99]	BMSCs; in vivo implantation	Intramembranous/woven bone	Dynamic GelMA/DNA dual-network hydrogel
Ju et al., 2026 [131]	BMSCs + HUVECs; nude mouse model	Osteogenic modules assembled into a vascularized graft	Bioprinted GDMA modules + bioadhesive modular assembly
Chen et al., 2026 [132]	Osteogenic/angiogenic cells; rat calvarial defect	Periosteal reconstruction	Bioprinted GelMA/SilMA + black-phosphorus nanosheets

BMSC, bone marrow-derived mesenchymal stromal/stem cell; DPSC, dental pulp stem cell; GDMA, gelatin methacryloyl/DNA-derived modular adhesive hydrogel; HUVEC, human umbilical vein endothelial cell; MSC, mesenchymal stromal/stem cell; pNIPAAm, poly(N-isopropylacrylamide); VEGF, vascular endothelial growth factor.

**Table 2B:** Representative studies: supporting lineages, self-organization and classification.

Study	Supporting lineages	Evidence of self-organization	Revised class
Kérourédan et al., 2019 [127]	Endothelial + host vascular cells	Patterned cells formed organized vessels in vivo; bone formation was host/scaffold guided	Engineered skeletal construct
Akiva et al., 2021 [128]	Osteoblast- and osteocyte-like states	Self-organized osteoblast-osteocyte network within endogenous mineralized collagen	Bone organoid
Park et al., 2021 [129]	Osteoclast precursors	Cells organized functionally, but lamellar architecture was inherited from the matrix	Engineered skeletal construct
Li et al., 2021 [109]	None added	Spheroid compaction and local ECM; no tissue-level hierarchy shown	Osteogenic spheroid
Liu et al., 2022 [110]	Host vascular cells	Self-assembly was limited to the spheroid unit	Osteogenic spheroid
Li et al., 2023 [130]	DPSC-derived endothelial-like cells	Scaffold-free construct retained lumen-like structures and mineralized matrix	Bone-like microtissue
Liu et al., 2024 [111]	Host macrophage and vascular responses	Spheroid-level assembly; function strongly instructed by nanomaterials	Osteogenic spheroid
Zhu et al., 2025 [99]	None added	Migration and self-organization generated spatiotemporal woven-bone architecture	Bone organoid
Ju et al., 2026 [131]	HUVEC-lined lumen + host arteriole	Self-organization within modules; tissue-scale geometry and perfusion were engineered	Bone organoid units in an engineered skeletal graft
Chen et al., 2026 [132]	Angiogenic niche + host vasculature	Osteogenic-angiogenic organization matured after 3D culture and in vivo conditioning	Periosteum organoid/engineered skeletal construct

**Table 2C:** Representative studies: matrix, remodeling and neurovascular evidence.

Study	ECM/mineralization	Remodeling	Vascular/neural integration
Kérourédan et al. [127]	New bone assessed in vivo; not a cell-derived organoid ECM	Not tested	Organized microvascular networks
Akiva et al. [128]	Cell-produced collagen mineralized under biological control; osteocyte lacuna-like embedding	No osteoclast-mediated resorption	Not included
Park et al. [129]	Osteoblast-deposited collagen/mineral distinguished from cell-free chemical mineralization	Stimulus-dependent osteoclastogenesis and localized remodeling	Not included
Li et al. [109]	Osteogenic markers and mineral deposition	Not tested	Host-associated vascularization
Liu et al. [110]	Osteogenesis and new-bone formation	Not tested	Host neovascularization
Li et al. [130]	von Kossa-positive mineral after sequential induction	Not tested	CD31-positive lumen-like structures; perfusion/anastomosis not shown
Liu et al. [111]	Osteogenic differentiation and defect mineralization	Host immune remodeling only	Host neovascularization; macrophage modulation
Zhu et al. [99]	Longitudinal mineralized-collagen hierarchy and matrix remodeling	Cell-mediated remodeling reported; coupled osteoclast resorption not shown	Not included
Ju et al. [131]	Acellular and uninduced controls; longitudinal collagen/mineral deposition	Remodeling pathways inferred; direct formation-resorption coupling not shown	Endothelialized lumen + host-vessel guidance; no neural component
Chen et al. [132]	Collagen-rich periosteal matrix, periostin and defect mineralization	Bone remodeling balance assessed in vivo	Stabilized microvasculature; no neural integration

**Table 2D:** Representative studies: mechanics, in vivo validation and limitations.

Study	Mechanical testing	In vivo validation/application	Major limitation
Kérourédan et al. [127]	Not reported	Mouse calvarial repair	Host healing and imposed pattern dominate; no post-print organoid maturation
Akiva et al. [128]	Controlled mechanosensitivity not tested	In vitro osteogenesis/disease model	Early woven bone only; no perfusion or coupled resorption
Park et al. [129]	Matrix stiffness measured; tissue mechanosensitivity not tested	In vitro remodeling model	Pre-existing bovine lamellae define structure; murine cells
Li et al. [109]	Not reported	Rodent bone repair	Phosphate/mineral-generating material can confound active mineralization
Liu et al. [110]	Not reported	Rodent bone repair/cell delivery	Drug and nanomaterial instruction dominate; tissue hierarchy not shown
Li et al. [130]	Not reported	In vitro regenerative/drug-development model	No in vivo validation, collagen hierarchy or remodeling assessment
Liu et al. [111]	Not reported	Rat calvarial repair	Exogenous dexamethasone/black phosphorus and host response dominate
Zhu et al. [99]	Hydrogel mechanics characterized; no controlled cellular load response	Four-week osseointegration	Material contribution to stiffness and mineral signal not fully separated
Ju et al. [131]	Compression compared with blank hydrogel and native tibia	Longitudinal in vivo maturation/transplantable graft	Requires host arteriolar insertion and in vivo maturation; not intrinsically perfused in vitro
Chen et al. [132]	Composite stability reported; cell-derived mechanics not isolated	Critical-sized rat calvarial repair	Models periosteum rather than complete bone; material and in vivo conditioning are substantial

“Not reported” indicates that the representative publication did not provide the listed evidence; it is not evidence that the biological property is absent.

## 6 Bioprinting as a tool for bone-organoid engineering

### 6.1 Major bioprinting modalities

Bioprinting is used in two conceptually distinct ways. “Printing organoids” deposits preformed organoid units whose identity has already been demonstrated, whereas “printing for organoid formation” deposits cells, spheroids or precursor tissues that may subsequently mature. The latter should not be called organoid printing unless post-print self-organization and bone-specific function are shown. Bioprinting can reproducibly place cell populations, materials, channels and interfaces, but geometric fidelity is a manufacturing endpoint rather than evidence of organoid maturation [131,[133], [134], [135], [136]].

The modalities below are therefore evaluated using the same parameters: resolution, printable viscosity, cell density, fabrication speed, injury mechanism, crosslinking, mineral compatibility, channel generation, scaffold-free use, scalability and direct evidence in bone organoids (Table 4A, Table 4BA and 4B). Technical ranges are platform-dependent and should be interpreted as typical operating envelopes rather than categorical advantages or limitations.

#### 6.1.1 Inkjet bioprinting: droplet-based deposition

Inkjet systems eject discrete droplets and can pattern low-viscosity cell suspensions or biomolecule-containing solutions with high positional accuracy [137]. Droplet formation depends on fluid properties, actuator energy, nozzle geometry and surface tension; Rayleigh-Plateau instability describes how a liquid jet breaks into droplets [138]. Inkjet printing is well suited to patterned delivery of cells, factors or mineral precursors, but its printable matrix range is constrained by narrow nozzles and low allowable viscosity. Cell concentration must also be controlled because sedimentation changes the number of cells delivered per droplet over time.

Thermal inkjet systems rapidly heat an element to generate a vapor bubble that ejects a droplet. Under optimized conditions, acceptable cell survival can be maintained despite brief temperature excursions [139,140]. Piezoelectric systems instead use actuator-generated pressure waves and do not rely on thermal actuation [141,142]. Neither approach is free from mechanical stress: acceleration, pressure, nozzle passage and repeated actuation may alter viability or function. For bone-organoid engineering, inkjet printing has a typical advantage in fine cellular patterning or gradient generation, but direct evidence of improved post-print skeletal organization or maturation remains very limited.

#### 6.1.2 Extrusion bioprinting: material versatility and cell density

Extrusion systems deposit continuous filaments using pneumatic, piston- or screw-driven pressure and can process a wide range of shear-thinning hydrogels and composite bioinks [143]. They are therefore the most widely used platform for bone-related bioprinting. This versatility creates a familiar trade-off: increasing viscosity or polymer concentration improves shape fidelity but also raises extrusion pressure, shear exposure and resistance to cell spreading. Reported parameters should include nozzle diameter, pressure or force, printing speed, temperature, cell density and the interval between deposition and crosslinking.

Pneumatic extrusion is simple and broadly compatible, although air compressibility can reduce response precision. Piston-driven systems provide more direct displacement, whereas screw-driven systems can process highly viscous or particle-filled inks at the cost of additional shear and mixing. Regardless of actuator type, shear-thinning behavior and rapid post-extrusion recovery are desirable because they reduce cell stress and help deposited filaments retain shape.

Nano-hydroxyapatite, bioactive glass, demineralized bone matrix, short fibers and other reinforcing components can be incorporated into extrusion bioinks [144]. These additives commonly improve stiffness or osteogenic signaling but may increase nozzle clogging, optical opacity, sedimentation and mineral-signal confounding. Their biological value should therefore be demonstrated against acellular, non-osteogenic and composition-matched controls (Table 3). Extrusion has the broadest direct relevance to bone constructs and emerging organoid-unit assembly, but evidence that it improves organoid maturation beyond geometry is still limited.

**Table 3:** Representative biomaterials relevant to bone-organoid engineering and regeneration.

Biomaterial category	Representative materials	Primary mechanism	Translational challenges	Commercial products	Clinical trial identifiers
Calcium phosphate ceramics [145]	Hydroxyapatite (HA), β-tricalcium phosphate (β-TCP), biphasic calcium phosphate (BCP)	Osteoconduction and mineral-phase mimicry	Brittleness, resorption mismatch and fatigue failure	Pro Osteon®	N/A
Bioactive glasses [146,147]	45S5, modified BG	Bioactive ion release supporting osteogenesis and angiogenesis	Brittleness, limited load-bearing capacity and degradation mismatch	NovaBone®	N/A
Natural & synthetic polymers [148,149]	Collagen, chitosan, PLA, PCL, PEG	ECM mimicry, cell adhesion and controlled delivery	Limited mechanics, degradation by-products and batch variability	FDA-approved PLA sutures, PCL implants	N/A
Biodegradable metals [[150], [151], [152]]	Mg-, Zn-, Fe-based alloys	Temporary structural support and ion-mediated bioactivity	Gas evolution, corrosion control and ion toxicity	MAGNEZIX®	N/A
Demineralized bone matrix(DBM)[ [153], [154]	DBM	Native ECM signaling and osteoinduction	Donor variability, residual immunogenicity and limited mechanical strength	Grafton® DBM	N/A
Composite scaffolds [[155], [156], [157]]	HA/polymer/cells	Combined osteogenic and mechanical functions	Interface instability, scale-up and reproducibility	N/A	NCT02609074
Growth factors & delivery systems [[158], [159], [160], [161]]	BMP-2, VEGF	Osteoinduction and angiogenesis	Burst release, short half-life and ectopic ossification	Infuse®,InductOs®	NCT01690260
Smart/immunomodulatory biomaterials [162]	IL-4 releasing systems, macrophage-polarizing scaffolds	Immune-microenvironment modulation	Spatiotemporal control and short cytokine half-life	N/A	N/A
Biofabrication/bioprinting inks [[163], [164], [165]]	GelMA, alginate, nanohydroxyapatite	Spatial control of cells, matrix and soluble cues	Trade-off between printability and cell viability	N/A	N/A
ECM-mimetic/organoid-compatible matrices [[166], [167], [168]]	Fibrin, hyaluronic acid, hybrid hydrogels	Support of self-assembly and maturation	Limited mechanics, variability and vascular integration	N/A	N/A

Despite its versatility, extrusion bioprinting faces several critical technical bottlenecks that constrain bone-organoid fabrication. First, achieving high cell densities while maintaining print fidelity remains a major challenge. Increasing cell density can alter bioink rheology and extrusion behavior, while the use of smaller nozzles or higher extrusion pressures increases cellular exposure to shear stress, creating a trade-off among cell density, viability and printing resolution [169]. Alternative fabrication strategies, including tomographic volumetric bioprinting and embedded or sacrificial printing within densely cellular matrices, can partly address this limitation by enabling rapid construct fabrication or internal channel formation without requiring the deposited bioink to support its own weight [170,171]. Second, multi-material printing with mineral-containing bioinks introduces additional complexities. The incorporation of hydroxyapatite, β-tricalcium phosphate or bioactive glass particles alters ink viscosity, yield behavior and mechanical properties, while large or aggregated ceramic particles may require larger nozzles and increase the risks of particle sedimentation, nozzle clogging and reduced filament resolution [172]. These problems can be mitigated by optimizing mineral-particle size distribution, solid loading, dispersion stability and the rheological properties of the surrounding shear-thinning polymer matrix. Third, the limited depth and spatial accuracy of photocuring constrain the use of DLP and stereolithography for thick, mineral-rich constructs. Hydroxyapatite particles scatter and absorb incident light, and variations in particle size distribution can reduce curing penetration while increasing unintended lateral curing, thereby generating heterogeneous crosslinking and dimensional inaccuracies. Particle size distribution, mineral loading, photoinitiator concentration, exposure dose and layer thickness should therefore be optimized together rather than independently. Addressing these bottlenecks requires integrated approaches that couple bioink chemistry, printing hardware and post-print culture conditions.

#### 6.1.3 Laser-assisted bioprinting (LAB): nozzle-free transfer

Laser-assisted bioprinting (LAB) uses a pulsed laser to generate a transient pressure event that transfers a small volume of cell-containing material from a donor layer to a receiving substrate [[173], [174], [175]]. The absence of a nozzle avoids clogging and enables micrometer-scale patterning of concentrated cell suspensions. LAB is potentially useful for arranging endothelial and osteogenic cells, but broader adoption is limited by equipment complexity, donor-ribbon preparation and throughput. In bone, direct evidence currently comes mainly from prevascularized engineered constructs, including in situ endothelial patterning that enhanced vascularization and repair, rather than from validated post-print bone-organoid maturation [127].

#### 6.1.4 Photocuring-based bioprinting: optical patterning

Stereolithography and digital light processing (DLP) solidify photocurable materials according to projected optical patterns [176,177]. Related DLP methods have also been used for high-resolution ceramic fabrication and for evaluating the printability of photocurable hydrogels [[178], [179], [180]]. Biological performance depends on photoinitiator chemistry, light wavelength, exposure dose, oxygen inhibition and optical attenuation by cells or mineral particles. These variables become particularly important in thick or highly filled bone bioinks.

In DLP, an entire projected layer is polymerized simultaneously, reducing fabrication time and improving in-plane resolution. Gelatin methacryloyl (GelMA) is widely used because it provides cell-adhesive motifs, supports enzymatic remodeling and crosslinks rapidly under suitable photochemistry [[181], [182], [183]]. The method can generate branching channels, trabecular-like voids and spatially separated cell compartments. However, increased GelMA concentration or light dose can restrict cell spreading and diffusion, while mineral particles can attenuate or scatter light. Direct bone-organoid evidence remains early; geometry and day-0 viability should not be treated as proof of later multilineage maturation.

Across modalities, direct evidence that printing improves organoid maturation remains substantially weaker than evidence that printing improves initial geometry (Table 4A, Table 4BA-4B). A valid comparison should test a printed construct against composition-matched, non-patterned or manually assembled controls and should assess delayed viability, lineage interaction, endogenous ECM organization, remodeling and functional performance after culture. Studies that print preformed organoid units should be distinguished from those that print cells or spheroids and only later claim organoid-like maturation.

**Table 4A:** Bioprinting modalities: operating ranges and injury mechanisms.

Modality	Resolution	Viscosity/cell density	Fabrication speed and principal injury mechanism
Inkjet	Typically 20–100 μm droplets	Usually <10–20 mPa s; commonly 10^^5^–10^^6^ cells/mL	High; Thermal pulse or piezoelectric acceleration; nozzle passage and sedimentation
Extrusion	Typically 100–500 μm filaments	∼30 mPa s to >10^^6^ mPa s; ∼10^^6^–10^^8^ cells/mL	Low–moderate; Pressure/shear, residence time and nozzle-wall stress
Laser-assisted	Typically 10–100 μm spots	Broad low–moderate viscosity; ∼10^^6^–10^^8^ cells/mL	Moderate; Laser energy, cavitation, jet impact and donor-ribbon effects
SLA/DLP	Typically 25–100 μm lateral; 10–100 μm layers	Photocurable resins, often <10 Pa s; ∼10^^6^–10^^8^ cells/mL	High by layer projection; Photoinitiator radicals, light dose, heating and optical attenuation

**Table 4B:** Bioprinting modalities: material compatibility, channels and direct bone-organoid evidence.

Modality	Crosslinking/mineral compatibility	Perfusable channels/scaffold-free use	Scalability and direct evidence
Inkjet	Usually secondary ionic/photo crosslinking; mineral particles increase clogging/sedimentation	Useful for channel lining or gradients; weak for bulk scaffold-free tissues	High for small patterns; direct bone-organoid evidence very limited
Extrusion	Ionic, thermal, enzymatic or photo crosslinking; best compatibility with mineral fillers	Sacrificial/coaxial channels feasible; spheroid/cell-dense printing feasible	Most scalable and most used in bone engineering; direct organoid-unit assembly is emerging
Laser-assisted	Crosslinking usually occurs after transfer; particulate loading complicates donor layers	High-resolution vascular patterns; nozzle-free and compatible with cell-only deposition	Equipment/throughput limit scale; bone evidence is mainly prevascularized engineered constructs [127]
SLA/DLP	Intrinsic photo crosslinking; mineral particles scatter light and reduce cure depth	Complex perfusable channels; scaffold-free/cell-only variants remain specialized	Strong geometric capability; direct proof of improved bone-organoid maturation remains limited

Ranges are illustrative rather than universal and depend on printer design, nozzle or optical settings, bioink formulation and construct scale. SLA, stereolithography; DLP, digital light processing. Evidence grading refers specifically to bone-organoid studies, not to conventional bone tissue engineering or unrelated organ systems.

### 6.2 Bioink selection: balancing printability and tissue maturation

Bioink design must balance immediate manufacturing requirements with long-term cell function. Natural polymers such as collagen, gelatin, fibrin, hyaluronic acid and alginate provide hydrated environments and, in some cases, cell-binding or degradable motifs. Synthetic or chemically modified components offer greater control over mechanics and crosslinking. Mineral fillers can support osteoconduction and nucleation, but excessively stiff or densely crosslinked inks may inhibit cell spreading and endogenous matrix assembly [184,185]. Composite GelMA bioinks containing mineral fillers or nanostructured calcium phosphate illustrate how printability and osteogenic support can be combined; nevertheless, over-crosslinking can compromise long-term spreading and matrix remodeling [186]. Print fidelity, swelling, degradation, diffusion and cell-mediated remodeling should therefore be assessed throughout the culture period.

### 6.3 CAD-driven strategies for spatial heterogeneity

Computer-aided design (CAD) can translate a biological hypothesis into a reproducible geometry [187,188]. A CAD model may specify pore size, channel branching, regional stiffness or the position of distinct cell populations, and patient imaging can define the external shape of a defect. Recent studies in biofabrication have demonstrated how geometric and material design parameters can be systematically optimized to improve biological performance and manufacturing fidelity [189,190]. Ju and colleagues developed the Neo-Organoid Visualization and Assembly (NOVA) strategy, which integrates bioprinting with hydrogel-based bio-adhesive assembly to enable high-throughput modular assembly of bone organoids [131]. Using CAD-guided design, this approach generates tissue-scale tubular bone-organoid grafts with in situ unidirectional guided vascularization, demonstrating that predefined channel architectures can direct endothelial organization and perfusable network formation [131]. Beyond geometric patterning, machine-learning approaches have also been explored for data-driven optimization of bioink properties and printing performance [190]. For example, Lee et al. used machine learning to identify relationships between bioink elastic modulus, yield stress, and printability, enabling the selection of formulations with improved shape fidelity [190]. Together, these studies illustrate that predefined geometry and data-driven optimization can improve control over the initial fabricated construct, whereas subsequent cellular organization and maturation ultimately shape the resulting tissue architecture [131,189,190]. The printed construct should nevertheless be treated as a starting condition rather than a final state: cells contract, migrate, deposit matrix and mineralize, while degradable materials change dimension. Validation should therefore compare the intended design with both the as-printed and matured constructs.

Pore gradients can balance cell retention, diffusion, vascular entry and mechanical stability [191]. Smaller pores increase surface area but may restrict infiltration, whereas larger interconnected pores improve transport at the expense of bulk strength [191]. Sacrificial inks, coaxial printing and hollow-channel molds can create immediately perfusable paths [192]. If the objective is to reproduce microvascular biology rather than simply deliver fluid, these conduits should be endothelialized or coupled to capillary sprouting [192]. Achieving endothelialization of perfusable channels can be accomplished through several complementary strategies. First, co-culture with endothelial cells (e.g., HUVECs or outgrowth endothelial cells) seeded onto channel luminal surfaces promotes the formation of a confluent endothelial monolayer that resists shear stress and supports barrier function [192]. Second, angiogenic factor delivery—including VEGF, angiopoietin-1 and FGF-2—can be spatially patterned within the hydrogel surrounding the channels to guide directional sprouting from the conduit into the adjacent matrix [193]. Third, perfusion conditions themselves play a critical role: controlled fluid shear stress (typically 0.5–5 dyn/cm^2^) upregulates endothelial nitric oxide synthase and promotes vascular stabilization, while intermittent perfusion may better mimic the dynamic mechanical environment of native bone [194,195]. These strategies are not mutually exclusive; combination approaches—for example, endothelial seeding followed by VEGF-gradient-guided sprouting under perfusion—are likely to yield the most physiologically relevant microvascular networks within bone organoids. Spatially varied materials can also model transitions between cartilage and mineralized bone or between marrow-like and cortical regions [191]. Interface integrity is therefore a critical readout because delamination or abrupt stiffness mismatch may dominate cellular responses. Multi-material or multi-nozzle printing can position BMSCs, chondrocytes, endothelial cells, neural cells and macrophages in distinct compartments; however, compartment boundaries should remain permissive to the intended direct contact, paracrine signaling or controlled migration rather than serve as visually complex but biologically isolated patterns [192].

### 6.4 Printing quality control and post-print validation

Bioprinting studies should distinguish manufacturing quality from biological quality. Manufacturing readouts include filament width, pore accuracy, layer registration, channel patency and dimensional change after crosslinking. Biological readouts include immediate and delayed viability, cell distribution, proliferation, differentiation, matrix deposition and remodeling. A construct that closely matches its CAD model on day 0 may subsequently fail, whereas a modestly off-target print may mature into a coherent tissue [196,197]. Both early and longitudinal assessments are therefore necessary. Reporting failed prints, batch yield and predefined exclusion criteria would substantially improve reproducibility.

## 7 Translational applications: from model systems to therapeutic constructs

The value of a bone organoid should be judged by the additional biological information or therapeutic function it provides for a defined task. Developmental and genetic disease modeling, metabolic and inflammatory disease, tumor–bone interaction, drug screening and regenerative grafts have different validation requirements and should not be evaluated with a single general framework (Fig. 7, Table 5A, Table 5BA and 5B).

**Fig. 7:**
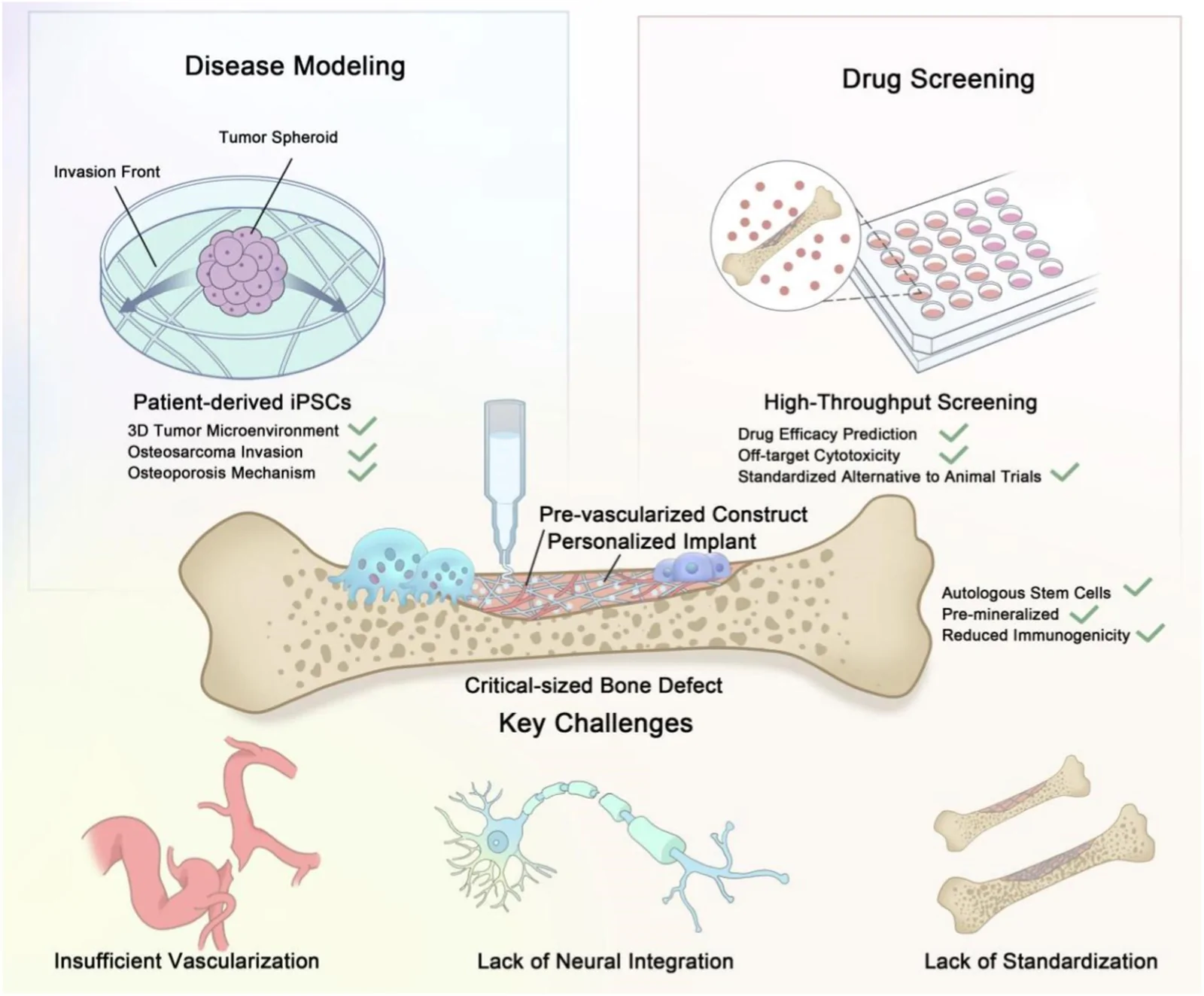
Translational applications and current challenges of bone organoids.

**Table 5A:** Application-specific objectives and required validation for bone organoids.

Application	Primary objective	Required validation
Developmental/genetic disorders	Reproduce developmental route and causal genotype	Stage-specific lineages, spatial ECM transitions, disease phenotype, rescue
Metabolic/inflammatory disease	Reproduce abnormal formation-resorption balance	Paired formation/resorption, osteocyte signaling, inflammatory/endocrine response
Tumor–bone interaction	Reproduce tumor behavior within a living skeletal niche	Clonal retention, invasion, osteolysis/osteogenesis, vascular/immune interaction
Drug screening	Generate reproducible, scalable response data	Batch CV, Z′ factor, dose-response, throughput, assay robustness, predictive validity
Regenerative graft	Restore integrated, mechanically functional bone safely	Donor/host contribution, perfusion, remodeling, mechanics, safety, manufacturability

**Table 5B:** Application-specific comparators, decision endpoints and evidence maturity.

Application	Essential comparator	Decision endpoint	Evidence maturity
Developmental/genetic disorders	Gene-corrected/isogenic control; patient tissue where available	Mechanistic fidelity	Emerging
Metabolic/inflammatory disease	Healthy/isogenic model; established perturbation	Dynamic remodeling phenotype	Early
Tumor–bone interaction	Matched patient tissue; standard therapy; tumor-only control	Patient-relevant progression and response	Emerging
Drug screening	Positive/negative controls; clinical or preclinical benchmark	Reliable rank-ordering or response prediction	Early
Regenerative graft	Acellular material; cells; spheroids; accepted graft standard	Durable structural and functional recovery	Preclinical

Bone organoids have potential applications in disease modeling, drug screening and regenerative graft development. Disease models should demonstrate genetic and phenotypic fidelity, screening platforms require reproducibility and predictive performance, whereas regenerative grafts should be evaluated for host integration, remodeling, mechanical recovery and safety. Appropriate controls—including conventional 2D cultures, acellular materials, dissociated cells, spheroids and, where applicable, autologous bone grafts or clinically accepted substitutes—are essential to determine whether organoid-level organization provides added value beyond its constituent cells and materials. Clinical translation remains constrained by insufficient vascular and neural maturation and by the lack of standardized fabrication, identity, potency and quality-control criteria.

### 7.1 Disease modeling and drug screening

Patient-derived iPSCs, primary tumor cells and genetically engineered skeletal cells can be combined with stromal, endothelial, immune and osteoclast-lineage populations to reproduce selected features of bone disease. Host cells and mineralized matrix influence invasion and treatment response in osteosarcoma and bone metastasis [198,199], while coupled formation and resorption provide more informative endpoints for metabolic bone disease than either process alone [200,201]. Recent advances have established specific bone disease models using organoid platforms. In bone tumor modeling, patient-derived chondrosarcoma organoids have been successfully established that faithfully recapitulate key histological and genetic features of parental tumors [202], enabling identification of SHH pathway activation driven by PTCH1 and BCOR alterations and demonstrating drug sensitivity to vismodegib. A biobank of 44 soft tissue and bone sarcoma organoid lines representing eight subtypes has been generated, providing a resource for personalized drug screening and precision immunotherapy [203]. In osteoporosis modeling, a three-dimensional vascularized and humanized bone-organoid model following endochondral ossification has been developed to recapitulate postmenopausal estrogen deficiency. This model revealed that estrogen withdrawal exacerbates formation of vessel-like structures (CD31^+^) and drives mineral deposition, providing mechanistic insights into bone mineral heterogeneity in osteoporotic bone that were not observable in non-vascularized models [204]. These examples demonstrate that incorporation of relevant supporting lineages—endothelial cells in osteoporosis models, tumor stroma in cancer models—is essential for reproducing disease-relevant phenotypes.

Human three-dimensional organization also alters drug penetration, cell state and survival signaling [205,206], potentially revealing responses missed in monolayer culture. Useful screening endpoints include region-specific viability, matrix formation or loss, osteoclast activity, vascular injury and recovery after treatment. High-throughput implementation will require miniaturization, automated imaging, standardized starting units and quality-control thresholds that exclude poorly formed organoids before analysis. The utility of bone organoids for drug testing is increasingly supported by concrete evidence. Organoid-based drug sensitivity testing has been successfully applied to chondrosarcoma organoids using the SHH pathway inhibitor vismodegib, demonstrating strong in vitro antitumor activity that correlated with genomic alterations. In sarcoma organoid biobanks, high-throughput drug screening has identified entity-specific sensitivities to various cytotoxic and targeted compounds, including MCL-1 inhibitors for CIC::DUX4 sarcomas [207]. Beyond oncology, patient-derived iPSC-based jawbone organoids have recapitulated phenotypic features of osteogenesis imperfecta, illustrating the potential of bone organoids for modeling genetic skeletal disorders and evaluating disease-specific phenotypes [208]. These studies highlight that three-dimensional organoid architecture alters drug penetration, cell state and survival signaling in ways that cannot be captured in monolayer culture, potentially revealing therapeutic responses—and resistance mechanisms—that would otherwise be missed.

### 7.2 Regenerative grafts

For regenerative applications, preconditioned cell-dense constructs may integrate more rapidly than dissociated cells delivered in a passive carrier [[209], [210], [211], [212]]. Current strategies aim to improve survival and host integration through prevascularization, endochondral priming, controlled mineralization and patient-specific geometry. Implantable organoids must also meet requirements that are less critical for laboratory models, including sterility, transport stability, surgical handling, predictable degradation and absence of unwanted proliferative cells. Specific examples of organoid-based grafts advancing toward clinical application include the NOVA strategy, which generates tissue-scale tubular bone-organoid grafts with in situ unidirectional guided vascularization suitable for surgical implantation in critical-sized bone defects [131]. Similarly, ossification center-like organoids (OCOs) comprising inner-core bone morphogenetic and neurotrophic spheroids generated via MSC-loaded 3D printing, alongside an outer-shell proangiogenic phase, have achieved fast bone-bridging and full-thickness reconstruction in calvarial defects [60]. Periosteum-derived organoids integrated with 3D-printed polycaprolactone scaffolds have also shown promise for enhanced craniofacial bone regeneration [213]. These examples illustrate that preconditioned, cell-dense constructs—particularly those incorporating prevascularization or endochondral priming—integrate more rapidly than dissociated cells delivered in passive carriers, though systematic comparison with autograft controls remains an essential next step.

Application-specific comparators are essential. Developmental or genetic disease models should be benchmarked against isogenic or gene-corrected controls and, where available, corresponding patient tissues. Osteoporosis models should include healthy or isogenic remodeling controls, whereas oncology organoids should be compared with matched patient tissues and standard therapeutic responses, with conventional 2D cultures or patient-derived xenograft models serving as complementary comparators where appropriate. For bone-defect repair, autologous bone graft remains the clinical reference standard; demineralized bone matrix (e.g., Grafton®) and calcium phosphate ceramics (e.g., Pro Osteon®) can serve as clinically relevant material comparators, alongside acellular materials, dissociated-cell constructs and spheroids as experimental controls. These comparisons help determine whether organoid architecture itself provides functional value beyond that of the constituent cells and materials.

## 8 Challenges and future perspectives

Bone-organoid engineering is advancing rapidly but remains at an early stage of standardization. Its major challenges are interdependent: increasing construct size worsens mass-transport limitations, stronger materials may impair cell function and adding cell types can improve biological relevance while reducing reproducibility.

### 8.1 Prevailing challenges

A central challenge is the rheological-biological trade-off in bioprinting: a material with excellent print fidelity may be too stiff, too slowly degradable or too stressful to extrude. A second challenge is incomplete vascular and neural integration. Perfusion channels can prevent gross necrosis but do not automatically generate a mature microcirculation or functional innervation. A third challenge is heterogeneity in cell source, spheroid size, matrix formulation, induction schedule and analytical endpoints. Without common identity, maturity and potency criteria, constructs carrying the same “bone organoid” label may represent fundamentally different biological systems [214]. From a quality-control perspective, reproducibility should therefore be evaluated at both the manufacturing and biological levels, including batch yield, organoid-size distribution, regional viability, matrix and mineral maturation, and application-specific functional readouts. Predefined inclusion/exclusion criteria and quantitative acceptance thresholds are also needed to distinguish acceptable constructs from poorly formed or incompletely matured organoids. Without such shared benchmarks, cross-laboratory comparison remains difficult and application-specific potency assays cannot be reliably transferred between studies. Long culture periods, mineral-related imaging artifacts and difficulty recovering cells for single-cell analysis further complicate comparison (Fig. 7).

### 8.2 Future directions

Progress will depend less on adding complexity indiscriminately than on controlling when and where complexity is introduced. Stimuli-responsive, enzymatically degradable or reversible matrices [215] can support printing and early consolidation and then progressively yield to endogenous ECM deposition and vascular invasion. Ideally, these transitions should be measurable and aligned with defined stages of organoid maturation, providing support when needed without preventing tissue remodeling.

Improving manufacturing precision will also be essential for producing organoids of consistent quality. Machine-learning methods can optimize printing parameters, classify construct quality from images and predict geometric changes during long-term culture. Wider use, however, will require standardized datasets, external validation and interpretable models. In the near term, automated microscopy coupled with quantitative morphological analysis is likely to provide greater practical value than fully autonomous scaffold design. Recent work on AI-enhanced 3D bioprinting for bone-organoid engineering has demonstrated that machine learning can optimize printing parameters, predict cell distribution and classify construct quality from image data. For example, AI-driven approaches have been applied to accelerate organoid optimization through data-driven parameter prediction, enabling more efficient experimental design and reducing iterative trial-and-error. The integration of artificial intelligence with bioprinting workflows is expected to enhance both manufacturing precision and biological reproducibility in bone-organoid production.

Once fabrication becomes more reproducible, the next objective is to reconstruct the physiological complexity of native tissue. Microfluidic platforms can provide controlled perfusion and mechanical stimulation and connect bone organoids with vascular, muscle, marrow, immune, liver or kidney modules. Such systems are valuable for studying systemic drug toxicity, metastatic colonization and endocrine regulation of bone. Maintaining compatible culture media and physiologically meaningful scaling across interconnected modules nevertheless remains a major technical challenge (Table 6).

**Table 6:** Comparison of experimental models used in bone-regeneration research.

Model system	Structural complexity	Vascularization	Mechanical fidelity	Microenvironmental fidelity	Future capability
2D Cell Culture [216]	Low	Low	Low	Low	Mechanistic screening
3D Spheroids [217]	Medium	Low	Low	Low–Medium	Multicellular maturation
Hydrogel Scaffolds [218,219]	Medium	Medium	Medium	Medium	Programmable niches
Animal Models [220]	High	High	High	High	In vivo validation
Ex Vivo Models [221]	High	Medium–High	Medium	High	Tissue-specific modeling
Organ-on-a-Chip [222,223]	Medium–High	Medium–High	Medium	Medium–High	Dynamic niche integration
Bone Organoids [12,224,225]	Medium–High	Medium	Low– Medium	Medium	Multilineage maturation
In Silico Models [226]	N/A	N/A	Simulated	Low–Medium	Predictive modeling

Table 6 Footnotes: Ratings refer to the current experimentally demonstrated performance of representative systems in the cited literature rather than their theoretical maximum capability. For structural complexity, Low indicates minimal tissue organization, Medium indicates three-dimensional but incompletely hierarchical organization, and High indicates well-developed multiscale tissue architecture. For vascularization, mechanical fidelity, and microenvironmental fidelity, Low indicates absent or limited reproduction of the corresponding feature, Medium indicates partial or engineered reproduction, and High indicates well-established or native-like reproduction. Intermediate ratings indicate performance between these categories. Future capability describes an anticipated direction rather than a currently established feature. These qualitative assessments are intended as approximate comparative guides rather than definitive rankings, given the substantial heterogeneity among individual experimental platforms.

As platforms become more sophisticated, standardized evaluation will become increasingly important. Consensus reporting should include cell source, passage number, organoid-size distribution, culture duration, matrix composition, induction schedule, regional viability, mineralization methods and mechanical properties. Potency assays should be application-specific: coupled remodeling for osteoporosis models, reproducible drug responses for screening platforms and vascularized bone formation for regenerative grafts. Reference materials and interlaboratory multicenter studies will be essential for regulatory acceptance and industrial translation.

To translate these general principles into actionable guidance, we propose the following minimum reporting checklist for bone-organoid studies, adapted from emerging consensus efforts in the field:

Cell source: species, tissue origin, isolation method, donor characteristics (age, sex, health status), passage number, culture medium and expansion conditions.

Organoid fabrication: initial cell density, matrix composition (including all components and crosslinking conditions), induction schedule (factors, concentrations, timing, duration), and assembly method (top-down, bottom-up or hybrid).

Maturation and culture: culture duration, medium composition and exchange frequency, oxygen tension, mechanical stimulation (if any), and perfusion conditions.

Characterization: organoid size distribution, viability (regional assessment, not just whole-construct averages), ECM organization (collagen deposition, spatial distribution), mineralization (method, timing, quantitative assessment), and cellular composition (lineage markers with spatial information).

Functional assays: mechanosensitivity (type and magnitude of stimulation, measured response), coupled formation/resorption (if applicable), and drug response (dose, exposure time, endpoints).

Quality control: batch-to-batch variability metrics, exclusion criteria for poorly formed organoids, and number of biological and technical replicates.

Additional reporting parameters: For multicellular or advanced bone-organoid systems, the minimum-information framework should additionally document lineage purity, cell ratios and assembly sequence, matrix manufacturer and lot information, manufacturing yield and the proportion of failed constructs, regional necrosis, vascular and neural characterization, mechanical properties and stimulation protocols, and predefined inclusion and exclusion criteria. Application-specific potency assays should also include predefined quantitative endpoints and acceptance criteria wherever feasible.

This checklist is intended as a living framework rather than a rigid prescription. It should be adapted to specific applications—for example, osteoporosis models would emphasize coupled remodeling assays, while screening platforms would prioritize reproducibility and throughput. Reference materials and future interlaboratory multicenter studies will be essential for validating these reporting standards and supporting regulatory acceptance.

Extensive characterization is required to determine whether engineered tissues faithfully reproduce native bone biology. Single-cell sequencing, spatial transcriptomics, proteomics, metabolomics and high-content imaging can define whether multiple lineages mature in appropriate spatial locations. These approaches are most informative when integrated with conventional histology, ECM analysis and functional testing. The goal is not simply to generate larger datasets, but to connect molecular states with tissue architecture and measurable biological function throughout organoid development.

### 8.3 Regulatory considerations for clinical translation

The clinical translation of bone-organoid-based therapeutics will require careful navigation of regulatory pathways that are still evolving [[227], [228], [229], [230], [231]]. Key regulatory considerations include:

Good Manufacturing Practice (GMP) compliance: Unlike laboratory-scale organoid production, clinical-grade bone organoids must be manufactured under GMP conditions with validated protocols for cell sourcing, expansion, differentiation, assembly and quality control [227,232]. This necessitates defined, xeno-free culture systems and traceable raw materials.

For implantable organoid products, sterility and biological safety should be evaluated according to the product composition and regulatory classification; where scaffold or device components are incorporated, biocompatibility assessment may additionally follow applicable ISO 10993 standards [232,233].

Potency assay development: A critical regulatory requirement is the establishment of potency assays that reliably predict therapeutic efficacy [234]. For bone organoids, this may include quantitative measures of osteogenic differentiation capacity, mineralization potential, vascularization competence and mechanical integrity—each of which must be validated against relevant clinical endpoints [224,234].

Path to first-in-human studies: Regulatory requirements for first-in-human evaluation of organoid-based products are jurisdiction dependent. In the European Union, certain cell- or tissue-engineered products may fall within Advanced Therapy Medicinal Product (ATMP) frameworks, whereas corresponding products in the United States and China are governed by their respective regulatory pathways [228,229]. Regardless of jurisdiction, preclinical development should address biodistribution, engraftment, ectopic tissue formation, immunogenicity, and long-term safety. Given the lack of standardized regulatory frameworks specifically for bone organoids, early engagement with relevant regulatory authorities through jurisdiction-specific consultation mechanisms is recommended to clarify product classification, manufacturing requirements, and clinical development plans [228,229,235].

Recent consensus efforts have begun to address these gaps by establishing standardization frameworks for bone-organoid development and application [224]. However, the field still lacks internationally harmonized guidelines that address the unique challenges of organoid-based therapeutics—including batch-to-batch variability, long-term stability and the demonstration of functional equivalence to native bone [[227], [228], [229], [230]]. Close collaboration among academic researchers, industry developers and regulatory bodies will be essential to establish robust, fit-for-purpose regulatory pathways that enable safe and effective clinical translation [[228], [229], [230]].

## 9 Conclusion

Bone organoids occupy a useful intermediate position between conventional osteogenic culture and whole-animal experimentation, but the term should be reserved for constructs that meet explicit biological criteria. The operational framework proposed here separates osteogenic spheroids, bone-like microtissues, scaffold-dominated skeletal constructs, bone organoids and high-fidelity bone organoids. Bioprinting can define initial geometry, cell placement and transport pathways, but it cannot substitute for post-print self-organization, active mineralization, remodeling or mechanobiological maturation. The next phase of the field should prioritize longitudinal, material-aware evidence and application-specific validation. Disease models should reproduce causal pathology and clinically relevant response; screening platforms should be reproducible and scalable; and therapeutic grafts should be safe, manufacturable and capable of durable host integration and mechanical recovery. No current representative model satisfies every high-fidelity domain, making transparent reporting of what was tested—and what remains untested—essential for progress.

## CRediT authorship contribution statement

**Yining Huang:** Writing – review & editing, Writing – original draft, Investigation, Formal analysis, Conceptualization. **Tianlong Zhang:** Writing – original draft, Investigation, Formal analysis. **Shuying Chen:** Writing – review & editing, Writing – original draft, Software, Formal analysis. **Hengbing Zhou:** Writing – review & editing, Writing – original draft, Formal analysis. **Haocheng Xu:** Writing – review & editing, Supervision, Formal analysis, Conceptualization. **Fan Zhang:** Writing – review & editing, Supervision, Formal analysis, Conceptualization. **Linli Li:** Writing – review & editing, Supervision, Funding acquisition, Formal analysis, Conceptualization. **Feizhou Lyu:** Writing – review & editing, Supervision, Resources, Funding acquisition, Conceptualization.

## Declaration of competing interest

The authors declare that they have no known competing financial interests or personal relationships that could have appeared to influence the work reported in this paper.

## Data Availability

No data was used for the research described in the article.
